# Modeling the Sequential Pattern Variability of the Electromotor Command System of Pulse Electric Fish

**DOI:** 10.3389/fninf.2022.912654

**Published:** 2022-06-28

**Authors:** Angel Lareo, Pablo Varona, Francisco B. Rodriguez

**Affiliations:** Grupo de Neurocomputación Biológica, Departamento de Ingeniería Informática, Escuela Politécnica Superior, Universidad Autónoma de Madrid, Madrid, Spain

**Keywords:** neural sequences, inter-pulse interval coding, temporal structure evolutionary tuning, computational neuroethology, multiple sequence network topology

## Abstract

Mormyridae, a family of weakly electric fish, use electric pulses for communication and for extracting information from the environment (active electroreception). The electromotor system controls the timing of pulse generation. Ethological studies have described several sequences of pulse intervals (SPIs) related to distinct behaviors (e.g., mating or exploratory behaviors). Accelerations, scallops, rasps, and cessations are four different SPI patterns reported in these fish, each showing characteristic stereotyped temporal structures. This article presents a computational model of the electromotor command circuit that reproduces a whole set of SPI patterns while keeping the same internal network configuration. The topology of the model is based on a simplified representation of the network with four neuron clusters (nuclei). An initial configuration was built to reproduce nucleus characteristics and network topology as described by detailed morphological and electrophysiological studies. Then, a methodology based on a genetic algorithm (GA) was developed and applied to tune the model connectivity parameters to automatically reproduce a whole set of patterns recorded from freely-behaving *Gnathonemus petersii* specimens. Robustness analyses of input variability were performed to discard overfitting and assess validity. Results show that the set of SPI patterns is consistently reproduced reaching a dynamic balance between synaptic properties in the network. This model can be used as a tool to test novel hypotheses regarding temporal structure in electrogeneration. Beyond the electromotor model itself, the proposed methodology can be adapted to fit models of other biological networks that also exhibit sequential patterns.

## 1. Introduction

Pulse mormyriforms, a group of weakly electric fish, produce electric pulses with high temporal precision. These fish have the ability to polarize their body in fast voltage transients whose deflection in the fish surroundings is detected by the fish using a specialized electric organ (Caputi, 1999). The electric organ discharges (EODs) occur as a result of the synchronous activation of modified muscle or nerve cells named electrocytes. This ability, known as active electroreception, is a well-suited sensory modality to study information processing in a living neural system. The signal from a freely-behaving fish can be monitored for long periods.

Information is encoded in the fish signal using a multiplexed temporal coding (Baker et al., 2013; Nagel et al., 2018). The pulse shape, with a mean duration of ~1 ms, is stereotyped, although there are variations among species (Hopkins and Bass, 1981), sex (Bass and Hopkins, 1983), or relative dominance (Carlson et al., 2000). The interval between EODs, known as the inter-pulse interval (IPI), is much larger and more variable than the duration of the EOD. At rest, IPIs are around 100–300 ms, but they fluctuate from less than 10 to more than 400 ms (Teyssedre and Boudinot, 1987). IPIs and sequences of pulse intervals (SPIs) are also relevant to information processing in these animals, as complex higher-level information can be encoded using this kind of temporal coding (Hopkins and Bass, 1981; Baker et al., 2013). For instance, IPIs decrease when the fish is actively probing their environment (von der Emde and Bleckmann, 1992). The timing flexibility of IPIs in this system gives rise to a remarkable set of SPI patterns with behavioral relevance, as we will discuss below.

**Figure 1:**
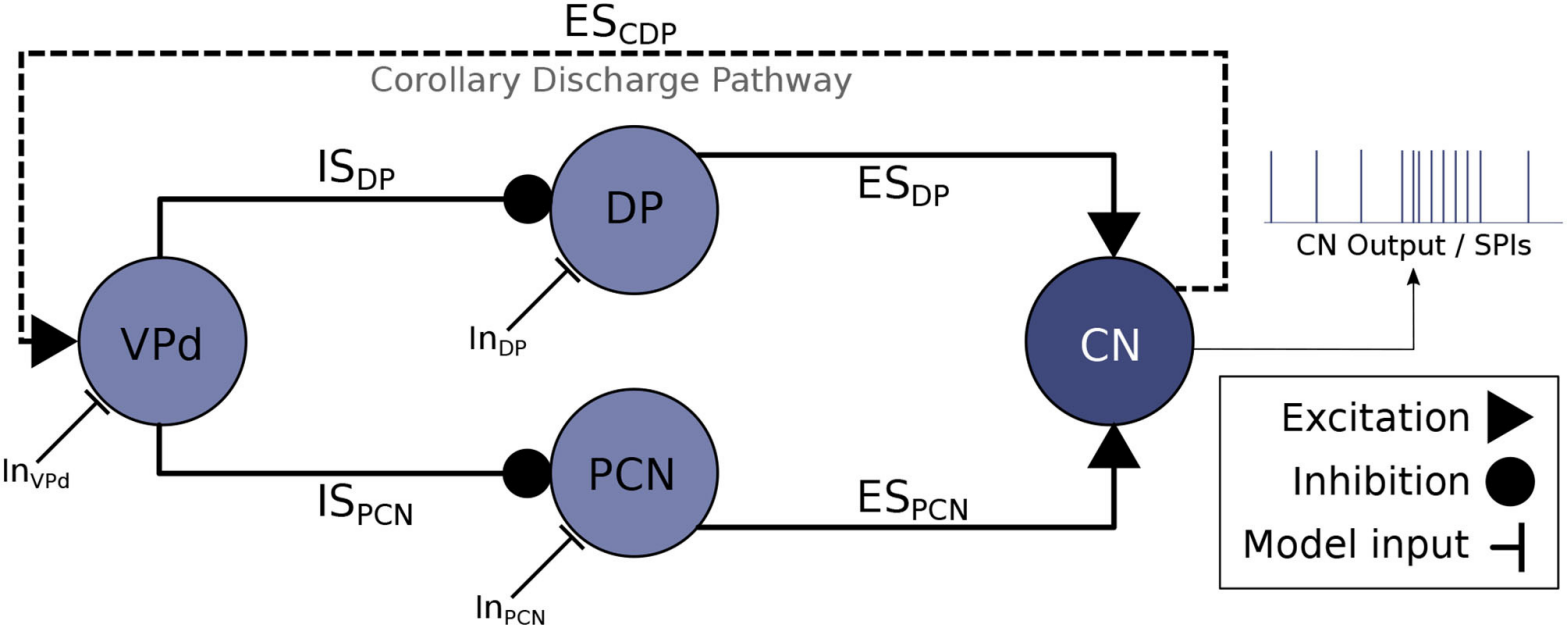
Abridged schematic of the electromotor command network based on Bell et al. (1983) and Carlson (2003) and used for developing the computational model discussed in this article. An EOD occurs after each action potential in CN (Grant et al., 1986), thus, CN activations represent the output of the model. CN receives excitatory projections from DP (ES_DP_) and PCN (ES_PCN_). Inhibitory afferents are driven to DP and PCN through VPd (IS_DP_ and IS_PCN_, respectively) triggered by the corollary discharge pathway (ES_CDP_), which makes VPd to fire a burst of action potentials right after the production of an EOD (Carlson, 2002b). This inhibition feedback seems to regulate the rhythm of EOD output (Von der Emde et al., 2000). Current inputs to the electromotor model (In_VPd_, In_DP_, In_PCN_) can be tuned to simulate different behavioral conditions that give rise to different SPIs.

The neural system responsible for controlling the timing of the EODs is the electromotor system, located in the central nervous system of the fish (Caputi et al., 2005). A neural ensemble known as the command nucleus (CN) initiates the EOD. Action potentials in CN are correlated with EODs (i.e., each action potential in CN leads to an EOD). Nevertheless, CN is not a pacemaker but an integrator system. It mainly receives synaptic input from the mesencephalic precommand nucleus (PCN) and the adjacent thalamic dorsal posterior nucleus (DP) (Figure 1). Nuclei DP and PCN receive projections from multiple sources, but they are both inhibited by the ventroposterior nucleus (VPd). The activation of VPd mediates this inhibition through a feedback mechanism, the corollary discharge pathway (Bell and Grant, 1989; Carlson, 2002b; refer to ES_CDP_ in Figure 1). Inhibition feedback is a mechanism for avoiding responses to the fish's own EOD and seems to regulate the resting electromotor rhythm (Carlson and Hopkins, 2004a). The IPIs of DP/PCN nuclei last, at least, as much as VPd bursts, which also emphasizes this fact (Carlson, 2003).

Recurrent inhibition is one shared feature with central pattern generators (CPGs), simpler neural control motor systems with rhythmic outputs (Selverston, 1999) which are well characterized in invertebrates. The intrinsic dynamics of the constituent neurons and the network topology of such CPGs are well-known. Using this knowledge, research on CPGs has taken advantage of computational modeling to explain how its rhythmic neural activity results from a combination of cellular and network properties (e.g., the swimmeret system of crayfish Sherff and Mulloney, 1996, the lobster stomatogastric ganglion Selverston, 2005, or the snail feeding network Vavoulis et al., 2007). In contrast to most CPGs, the specific dynamics of the nuclei and the synaptic properties within the electromotor system for reproducing the variability of SPIs shown by this system are not known. Furthermore, the electromotor system does not produce rhythmic outputs but a variety of SPI patterns with characteristic temporal structure and behavioral significance.

We have developed a model of the electromotor command system of pulse mormyrids capable of reproducing the variability of temporal firing patterns shown by these fish as a function of the input while sustaining the same network architecture. The model topology and nucleus dynamics are based on the results from previous physiological studies of the pulse mormyrid electromotor system (Bell et al., 1983; Carlson, 2002b, 2003; Carlson and Hopkins, 2004a).

Samples of these patterns were obtained for this work from experimental data recordings of living *Gnathonemus petersii* specimens (Forlim and Pinto, 2014; Forlim et al., 2015; Lareo et al., 2016, 2017) for the first time. These types of patterns have been previously characterized in other species of the *Mormyridae* family (Carlson and Hopkins, 2004b).

An automated method based on genetic algorithms (GA), with the development of a multiobjective fitness function for this system, was applied for synaptic parameter setting. Patterns recorded from *G. petersii* were used to fit the model parameters. All of the previously described SPI patterns with behavioral significance were consistently reproduced. Finally, the robustness of the model was tested under systematic variations of network inputs to assess the validity and discard overfitting to these inputs. We argue that the proposed methodology to generate functional temporal structure in SPI patterns can be applied to model the electromotor command network in a variety of fish species. Even more, the methodology proposed in this study can be generalized to fit models of other biological networks that also exhibit sequential patterns.

## 2. Computational Model of the Electromotor Command Network

Figure 1 illustrates the model of the electromotor command network described in the following subsections. We also describe below the genetic approach used to fit the synaptic connectivity parameters that give rise to distinct activity patterns as a function of the stimuli.

### 2.1. Characteristic Sequences of Pulse Interval

Pulse mormyrids generate a wide variety of electrical activity patterns using different sequences of pulse intervals (SPIs) and resting IPIs range from ~100 to 300 ms. Previous studies have described several stereotyped SPIs related to typical social behaviors (Carlson and Hopkins, 2004b). Accelerations, scallops, rasps, and cessations are four relevant SPIs that have been described (Carlson and Hopkins, 2004b; Caputi et al., 2005). The temporal structure of SPIs (i.e., the timing of the spikes and the relative change between IPIs) is the defining part of these behavioral displays. Carlson and Hopkins (2004b) demonstrated the existence of three different modal classes of SPI displays (accelerations, scallops, and rasps) with an increased firing rate that differs categorically in their temporal structure. As scallops are defined by a pause in the EOD generation, they also define a fourth categorically different display (Moller, 1970; Moller et al., 1989; Kohashi et al., 2021).

**Figure 2:**
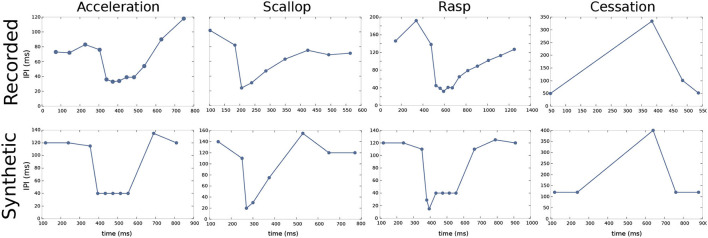
Characteristic SPI patterns recorded from freely-behaving *Gnathonemus petersii* specimens (top) and examples of corresponding synthetic SPIs to evolve and validate the electromotor command network model (bottom). Regarding the different SPIs, accelerations are sustained IPI shortenings to a series of almost regular shorter IPIs; scallops are sudden drops to very short IPIs (below the minimum IPI in accelerations) followed by a recovery to regular resting IPIs; rasps are a combination of a sudden drop to very short IPIs as in a scallop followed by a step increase of IPIs duration for a sustained series of IPIs; cessations are activity dropping in the EOD generation for periods up to 1 s. All of them have different temporal structures (Moller, 1970; Moller et al., 1989; Carlson and Hopkins, 2004b), different behavioral significance, and they result from different activations within the electromotor network (Carlson, 2003; Carlson and Hopkins, 2004a). Recorded SPIs were obtained from experimental data recordings of living *G. petersii* specimens and were used as targets for the R-GA configuration of the model. A set of synthetic SPIs were constructed preserving the characteristic temporal structure of previously reported SPI patterns (Carlson and Hopkins, 2004b), they were used as targets for obtaining the S-GA configuration of the model.

Accelerations are sustained IPI shortenings to a series of almost regular shorter IPIs. Accelerations (refer to Figure 2) are related to the activation of the neural ensemble known as the adjacent thalamic dorsal posterior nucleus (DP, refer to Figure 1; Carlson and Hopkins, 2004a). According to Kramer (1976), this kind of SPI is related to aggressive behaviors.

Scallops (refer to Figure 2) are sudden drops to very short IPIs (its minimum IPI use to be below the minimum IPI in accelerations) followed by an almost immediate recovery to regular resting IPIs. Contrary to what occurs in accelerations, IPIs drops in scallops are not sustained, so the total duration of the display is shorter than accelerations or rasps. A scallop pattern in CN takes place after the activation of the mesencephalic precommand nucleus (PCN, refer to Figure 1). This kind of firing pattern may serve as an advertisement signal (Serrier and Moller, 1989; Caputi et al., 2005).

Rasps (refer to Figure 2) are a type of IPI pattern that has an initial sudden decrease to very short IPIs, similar to the one observed in scallops, followed by a step increase of IPIs duration for a sustained series of IPIs, like a long tail of short regular IPIs similar to the ones observed in the acceleration pattern (Hopkins, 1981). Rasps likely rely on activation of both DP and PCN (Caputi et al., 2005) (refer to network model in Figure 1). This SPI is evoked during male courtship behavior (Carlson and Hopkins, 2004b).

Finally, cessations (refer to Figure 2) correspond to activity dropping in the EOD generation for periods up to 1 s. A cessation is evoked by the activation of the ventroposterior nucleus (VPd, refer to Figure 1). Submissive behavior has been associated with this SPI (Moller, 1970; Moller et al., 1989), and it has also been related to increased sensitivity to receiving communication signals (Kohashi et al., 2021). We will use these four representative patterns to validate our electromotor network modeling approach.

All four SPI displays (accelerations, scallops, rasps, and cessations) are well-defined behavioral firing patterns, although they show a large temporal variability (both in its constituent IPIs and their overall duration) not only between patterns but also between samples of the same pattern. Examples of this variability in SPI displays, especially regarding accelerations, are shown in Carlson and Hopkins (2004b). Also, previous studies describing acceleration-like bursts discuss timing variations (Bell et al., 1974; Kramer, 1976). Regarding cessations, Moller et al. (1989) show variations in the mean duration of silences relating them to fish size. Both recorded and synthetic SPI datasets used for GA optimization were constructed to reflect this variability.

### 2.2. Nuclei Model

Four different neuron ensembles in the electromotor command chain were modeled. They were the medullary command nucleus (CN), the mesencephalic precommand nucleus (PCN), the adjacent thalamic dorsal posterior nucleus (DP), and the dorsal region of the ventroposterior nucleus (VPd). Each nucleus was simulated using the neuron model developed by Izhikevich (2003). This simplification was adopted due to the lack of biological details both in the constituent neurons and the synaptic properties at the nucleus level. This model combines biological plausibility and computational performance characteristics of the integrate-and-fire neuron modeling approach (Long and Fang, 2010). It is based on a two-dimensional system of ordinary differential equations:


(1) $$\begin{array}{l} \frac{dv}{dt}\left(t\right)=0.04v^{2}+5v+140-u+i_{syn}\left(t\right), \end{array}$$

(2) $$\begin{array}{l} \frac{du}{dt}\left(t\right)=a\left(bv-u\right), \end{array}$$

with an auxiliary after-spike resetting:


(3) $$\begin{array}{l} \text{if}v\geq 30\text{mV, then }\{\begin{array}{c} v=c\\ u=u+d, \end{array} \end{array}$$

where *v* represents the neuron's membrane voltage and *u* is a voltage variable representing the combined action of ionic current dynamics. The parameters *a*, *b*, *c*, and *d* set the working regime of the neuron model. *i*_*syn*_ is the model external input.

A wide range of neuron dynamics, and in particular firing temporal structures, can be reproduced by selecting different values of the parameters *a*, *b*, *c*, and *d*, as shown in Izhikevich (2003).

The parameters of the neuron model were first adjusted to reproduce the dynamics of the nuclei described by previous neurophysiological studies of the electromotor command network. According to Carlson (2003), units from DP/PCN nuclei showed wide variations in baseline frequency, from sporadical firing units to units with high spiking rates, so no baseline firing frequency was preselected for the DP/PCN models. Nevertheless, as it occurs in the living network, a bimodal structure arose from the IPIs intervals before and after CN action potential in both DP/PCN, with larger intervals occurring after the CN activation. VPd nucleus model fires high-frequency sequences of action potentials with a noticeable spike frequency adaptation, which is in accordance with physiological records showing bursts of action potentials from VPd during each IPI. This behavior was modeled using a *low-threshold spiking* firing regime, which is usually displayed by inhibitory neurons (initial synaptic parameters were also adjusted to reproduce this behavior). Model parameters were also selected to ensure that the intrinsic dynamics could reproduce the characteristic temporal structure observed in the resulting pattern evoked after activation of each nucleus (i.e., frequency adaptation in PCN, regular frequency in DP, bursting behavior in VPd, and spike integration in CN). This was an assumption based on physiological results in network behavior. However, the intrinsic characteristics of the neurons in each nucleus are not known in detail, neither under spiking nor step function inputs. Also, there was considerable variation in the timing of the first spike of the burst relative to CN, intraburst firing rate, burst duration, and the number of spikes per burst from VPd units, so none of these characteristics was *a priori* selected. Finally, the CN model was configured to a *phasic spiking* firing regime as it mainly integrates inputs from DP and PCN (Carlson, 2002a).

The parameter values for modeling each nucleus are listed in Supplementary Section 2 and Supplementary Table S2 depicts the activity of each model nucleus (isolated from the network) in response to a step current input, and also an example of the response of each model nucleus to synaptic input from the network model.

### 2.3. Synapses Model

We reproduced neural projections using a model of chemical synapses as chemical inter-nuclei communication takes place in the real electromotor command network. When the pre-synaptic target generates an action potential in these synapses, a certain amount of neurotransmitters are released and bind to the postsynaptic receptors. The mathematical description used to model this behavior is based on a description of the receptor bindings (Destexhe et al., 1994). These equations define a simple method for computing synaptic currents with low computational cost. The ratio of bound chemical receptors in the post-synaptic target (*r*) during a pulse (*t*_*f*_ < *t* < *t*_*r*_) and after the pulse (*t*_*r*_ < *t*) was calculated as follows:


(4) $$\begin{array}{l} ṙ=\{\begin{array}{cc} \alpha \left[T\right]\left(1-r\right)-\beta r, & \text{if }t_{f}<t<t_{r}\\ -\beta r, & \text{otherwise}, \end{array} \end{array}$$

where α and β are the forward and backward rate constants for transmitter binding and [T] is the neurotransmitter concentration.

The beginning of a pulse (*t*_*f*_) was detected when the presynaptic neuron's membrane potential crossed a given *threshold*. The time between *t*_*f*_ and *t*_*r*_ was defined as the maximum release time (*t*_*max*_). Both *threshold* and *t*_*max*_ were tuned as a parameter of the synapses (refer to Table 1).

**Table 1:** Relevant synaptic parameters of the GA adjusted to recorded *Gnathonemus petersii* patterns (R-GA, refer to Figure 2) configurations of the model (*threshold* = 0; *E*_*syn*_ = −80; T = 1; refer to Equations 4, 5).

**R-GA**				
**Synapse**	**α**	**β**	***g*_*syn*_**	***t*_*max*_**
IS_*DP*_	0.539	5.297e-3	-1.658e-1	177.288
IS_*PCN*_	5.948	1.295e-3	-3.077e-1	167.175
ES_*DP*_	5.982	1.200e-1	2.381e-1	9.51458
ES_*PCN*_	5.027	2.186e-1	1.997e-1	84.4537
ES_*CDP*_	4.433	1.371e-2	6.471e-1	428.988

From the ratio of bound receptors is given by Equation 4, the current received by the post-synaptic target, *i*_*syn*_, at any time *t* was then calculated as follows:


(5) $$\begin{array}{l} i_{syn}\left(t\right)=g\cdot r\left(t\right)\cdot \left(V_{\text{post}}\left(t\right)-E_{\text{syn}}\right), \end{array}$$

where *g* is the synaptic conductance, *V*_post_(*t*) is the post-synaptic potential at time *t*, and *E*_syn_ is the synaptic reversal potential at the same time.

The topology of the model was set up using a standard configuration of the model of chemical synapses (ES_DP_, ES_PCN_,IS_DP_, IS_PCN_, ES_CDP_ in Figure 1). The adjustment of the parameters of these synapses is essential to generate the four types of SPI patterns exhibited by the electromotor command network. An *ad hoc* iterative tuning of the parameters of the model was performed to match previously described dynamics in the electromotor command network: (i) DP/PCN units firing sporadically before CN, (ii) DP/PCN remaining silent for tens to hundreds of milliseconds after an action potential from CN, and (iii) VPd firing a burst of action potentials during DP/PCN silence starting ≈1–8 ms after CN activation.

The inherent complexity of tuning all the parameters to reflect all these dynamics shown by the real network led to the development of an automatic method for tuning synaptic parameters of the model. Instead of making a purely random search in the parameter space, this initial *ad hoc* configuration served as a biologically plausible starting point. This method is described in the next section.

### 2.4. Automatic Selection of Synaptic Parameters

An automatic approach was used to tune the parameters of the model synapses. This approach was followed to overcome the lack of specific physiological information about the characteristics of the synaptic configuration in the real system. A genetic algorithm (GA) was developed and applied to evolve the synapses' parameters to reproduce the variability of the electromotor command system patterns. Both synthetic and recorded sets of patterns were used during GA optimization, resulting in two different configurations of the model: S-GA (optimized to synthetic patterns) and R-GA (optimized to recorded patterns).

Each individual *I* in the population of the GA was conformed by a set of 20 parameter values: α, β, *g*, *t*_*max*_ for each of the 5 synapses of the model (refer to Figure 1, Equations 4, 5). Each *I* in a generation had different randomly modified values from the initial configuration.

Each generation, starting from the initial one, was formed by 100 different individuals. The GA follows a steady-state GA scheme (Agapie and Wright, 2014; Lareo et al., 2018). In this scheme, a temporary population was created by cross and mutation. It was added to the original population. All individuals were then evaluated (using the fitness function described in the next section) and ranked according to their grades. The worst individuals were discarded in order to return the population to its original size. The best individuals (10%) were maintained between generations. This process continued for a predefined number of generations or until a relative increase of the initial fit was reached (refer to Supplementary Section 4 for more details).

As usual, when using GAs for optimization, a balance is needed between the parameter space size and the execution time needed to cover it appropriately. In order to achieve this balance, multiple runs of the GA were performed with different narrower or wider ranges of valid parameter values (refer to Supplementary Table S8 for details of different runs of the GA).

#### 2.4.1. Fitness Function

Each individual, defined by a set of values for the previously specified parameters, was evaluated being simulated under a predefined set of 4 different simulation cases (*S*), each one corresponding to a target SPI: Acceleration (*S*_*acc*_), scallop (*S*_*sca*_), rasp (*S*_*rasp*_), cessation (*S*_*cess*_). Simulation cases (*S*_*pat*_) established the current inputs required to reproduce the *pat* pattern. Each individual *I* was modeled under all four simulation cases. The fitness function of the overall individual (*f*(*I*)) was defined as the sum of the fitness results under each case:


(6) $$\begin{array}{l} f\left(I\right)=f_{acc}\left(I\right)+f_{sca}\left(I\right)+f_{rasp}\left(I\right)+f_{cess}\left(I\right). \end{array}$$

**Figure 3:**
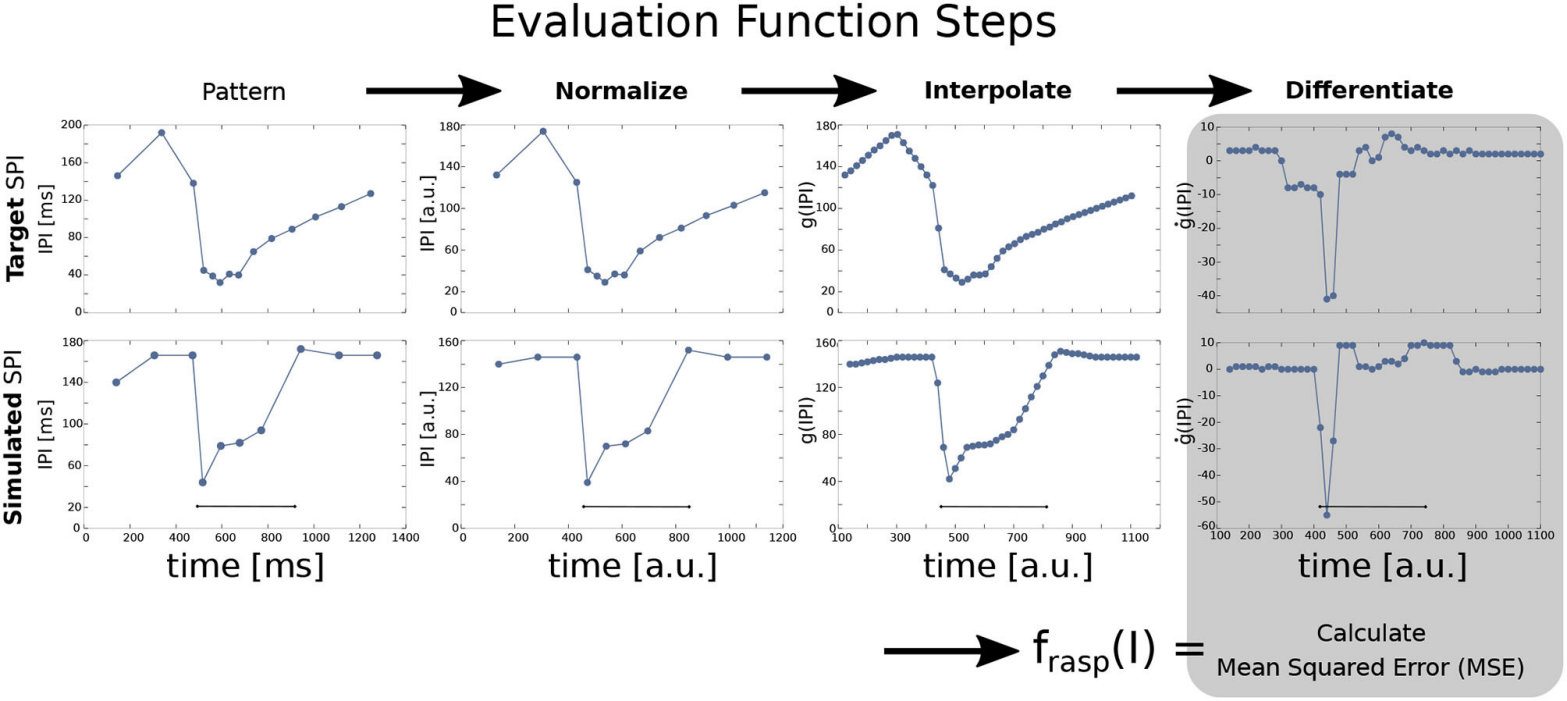
Steps for SPI output fitness evaluation in a rasp pattern example comparing a target SPI (top) and a simulated SPI (bottom). The input current step function is injected at 500 ms and lasts for 400 ms. First, the SPI is normalized to 1,000 arbitrary units (in the figure, it is represented starting at the first IPI). Normalized IPIs are then interpolated every 20 ms and differentiated (ġ(*IPI*)). Finally, the mean squared error (MSE) is calculated between the target SPI pattern and the simulated one to get the fitting value for one specific pattern (Equation 7). The fitness value of the overall model is the sum of the fitness results for each pattern (refer to Equation 6).

The four target patterns (acceleration, scallop, rasp, and cessation) were defined in terms of an ordered sequence of IPIs (*p*_0_, ..., *p*_*m*_) where *p*_*i*_ is each IPI arranged successively. The output of a simulation case was also defined in terms of an ordered sequence of IPIs. Sequences were normalized to the same duration (1,000 arbitrary units) and regularly interpolated (every 20 arbitrary units, obtaining *n* = 50 points) to detect the pattern shape. This was done to compare and subsequently differentiate them. The SPI patterns are better defined by the increasing/decreasing slopes between IPIs better than the absolute timing values (refer to Figure 3 for an example). Nevertheless, for each SPI the timing of the inputs and the total simulation times are fixed, so even though differentiated data is relative, it contains information about the absolute value of the IPIs. Previous versions of the GA were based on a fitness function using exclusively absolute IPI values (refer to Lareo et al., 2018) thus obtaining worse results. The pseudocode of this fitness function is shown in Algorithm 1.

**Algorithm 1:** Pseudocode of the fitness function, which relies on three different procedures: First, *tranformSPI* procedure considers the list of sequential IPIs that form one SPI and applies the transformations showed in Figure 3. Then, *evalPattern* procedure applies *tranformSPI* to both the simulated SPI (*simulSPI* procedure) and the target pattern (*targetSPI*) to compare them by calculating the mean squared error (MSE) between both (*msePattern*, calculated as described in Equation 7). The *evalPattern* procedure returns the fitting value of a pattern (*fitValuePattern*) calculated as described in Equation 8. Finally, *evaluate* procedure invokes *evalPattern* for each of the four patterns (scallop, acceleration, rasp, and cessation) and adds them to return the fitness value of the overall individual (*fitValue*, equivalent to *f*(*I*) in Equation 6).

**procedure** transformSPI(*SPI*)
SPI ← normalize(*SPI*)
SPI ← interpolate(*SPI*)
SPI ← diff(*SPI*)
**return** *SPI*
**end procedure**

**procedure** evalPattern(*I*,*S*_*pat*_)
transfSimulatedSPI ← transformSPI(simulate(*I*, *S*_*pat*_))
**for** *targetSPI* in *targetSPIs*(*S*_*pat*_) **do**
transfTargetSPI ← transformSPI(targetSPI))
*msePattern*←*min*(msePattern,
MSE(*transfTargetSPI, transSimulSPI*))
**end for**
*fitValuePattern*←1/(1+*msePattern*)
**return** *fitValuePattern*
**end procedure**

**procedure** evaluate(*I*)
*fitValue*←0
**for** *S*_*pat*_in*Simulations* **do**
*fitValue*←*fitValue*+evalPattern(*I, S*_*pat*_)
**end for**
**return** *fitValue*
**end procedure**

Finally, the fitting value *f*_*pat*_(*I*) was calculated by comparing the target SPI patterns after these transformations ($p_{0}^{t},...,p_{n}^{t}$) with the SPI model outputs after the same transformations ($p^{S}{\left(I\right)}_{0},...,p^{S}{\left(I\right)}_{n}$) using the mean squared error (*MSE*) as follows:


(7) $$\begin{array}{l} MSE\left(I\right)=\frac{\sum_{i=0}^{n}{\left(p_{i}^{t}-p^{S}{\left(I\right)}_{i}\right)}^{2}}{n}, \end{array}$$

(8) $$\begin{array}{l} f_{pat}\left(I\right)=\frac{1}{1+MSE\left(I\right)}. \end{array}$$

Mean squared error (Equation 7) was calculated for all target SPI pattern examples (note that several examples were provided for each pattern). Then, only the target example with the minimum MSE (i.e., the one which is closest to the simulated SPI output) was considered to calculate *f*_*pat*_(*I*) for each pattern (Equation 8 and EvalPattern procedure in Algorithm 1). After *f*_*pat*_(*I*) was calculated for each pattern this way, the global fitness function was calculated as defined in Equation 6.

#### 2.4.2. Model Simulation

**Figure 4:**
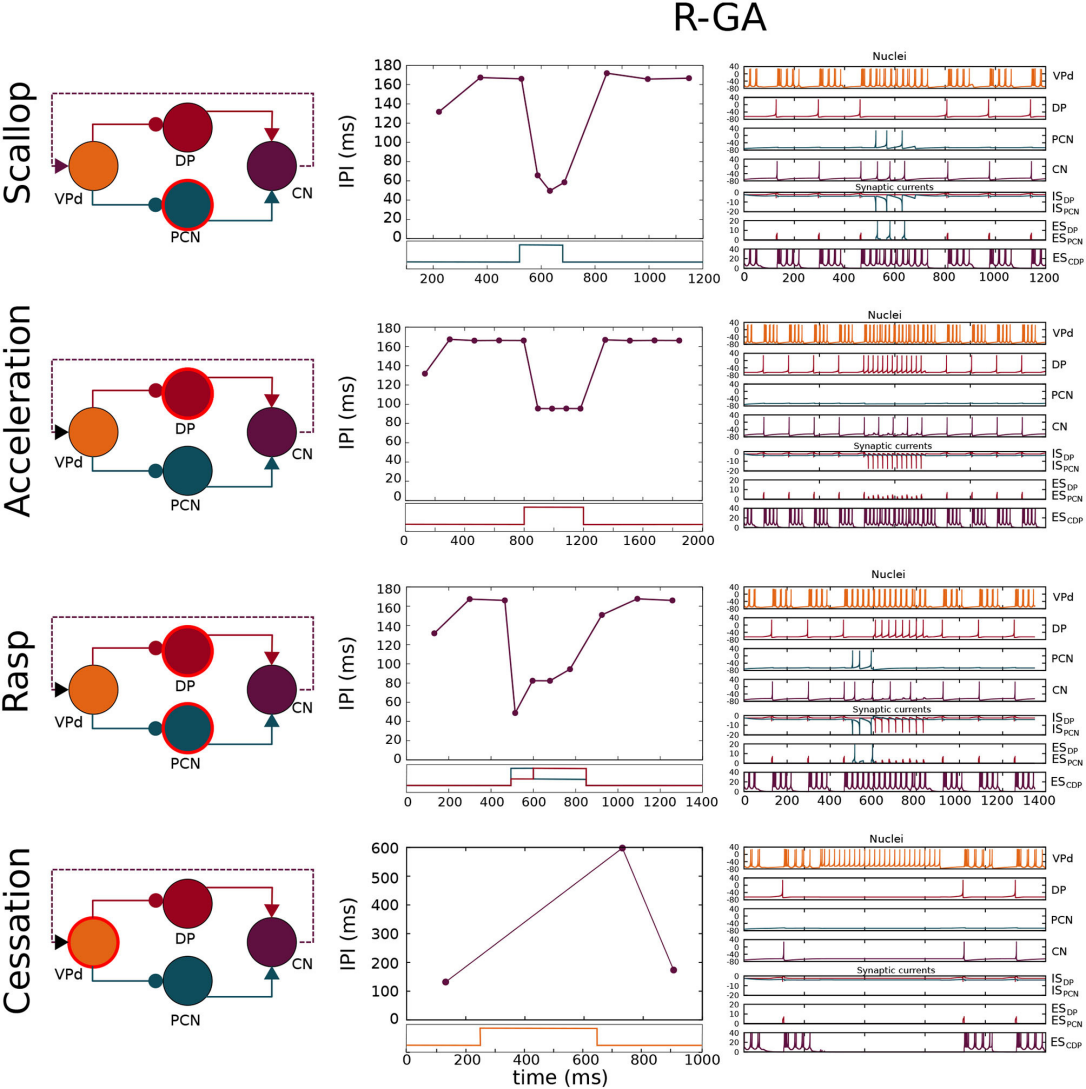
Simulation of the four SPI patterns in the configuration adjusted to patterns recorded from freely-behaving *G. petersii* specimens (R-GA). Each row shows a schematic of the network where the nucleus/nuclei responsible for generating the SPI (Caputi et al., 2005) is/are highlighted using a circular red stroke. The results are depicted in two columns: the first one shows SPIs resulting from the simulation, and the second one shows the nuclei voltages and synaptic currents. Each chart is related to its corresponding nuclei/synapse by color. Step functions used as current inputs to activate nuclei/nucleus are depicted under each SPI pattern, and they are also related to their corresponding nuclei by color. Relevant model parameters optimized by the GA are described in Table 1. Simulation parameters used in the simulations are described in Supplementary Section 3.

Since the model presented here is multi-objective (i.e., it reproduces different SPI patterns when modifying only the model inputs), a set of four different simulation cases (each one related to a distinct SPI pattern) was determined. Each simulation case was defined by the input current values received by the model (In_VPd_, In_DP_, In_PCN_ in Figure 1) during the simulation of the four SPI patterns shown in Figure 4.

These predefined inputs were step-functions representing projections received by VPD, DP, and PCN nuclei from other nervous system sources. We assume that with no input, there is no activity in the model network. Before each simulation, the model was initialized for a random amount of time. During this period, the model is stimulated to reproduce a base rhythm of IPIs of ~120 ms (in the range of IPIs shown by the real fish during resting). Input values for each SPI case are described in Supplementary Section 3. The source code of both the electromotor command network model and the GA for synaptic parameter optimization are provided (refer to Supplementary Section 13). The GA can be easily adapted to any alternative experimental data from weakly electric fish.

#### 2.4.3. Robustness to Input Variability and Overfitting Analysis

Analysis for all the different configurations of the model was conducted to assess robustness to input variability and discard overfitting to the predefined stimulation cases. The step functions used as inputs in the simulations are meant to reproduce the nuclei activation associated with each SPI (refer to Supplementary Section 3). Consequently, similar SPIs were expected to result from distinct inputs as long as the appropriate nucleus was stimulated. According to this hypothesis, a robustness analysis of input variability was conducted.

**Figure 5:**
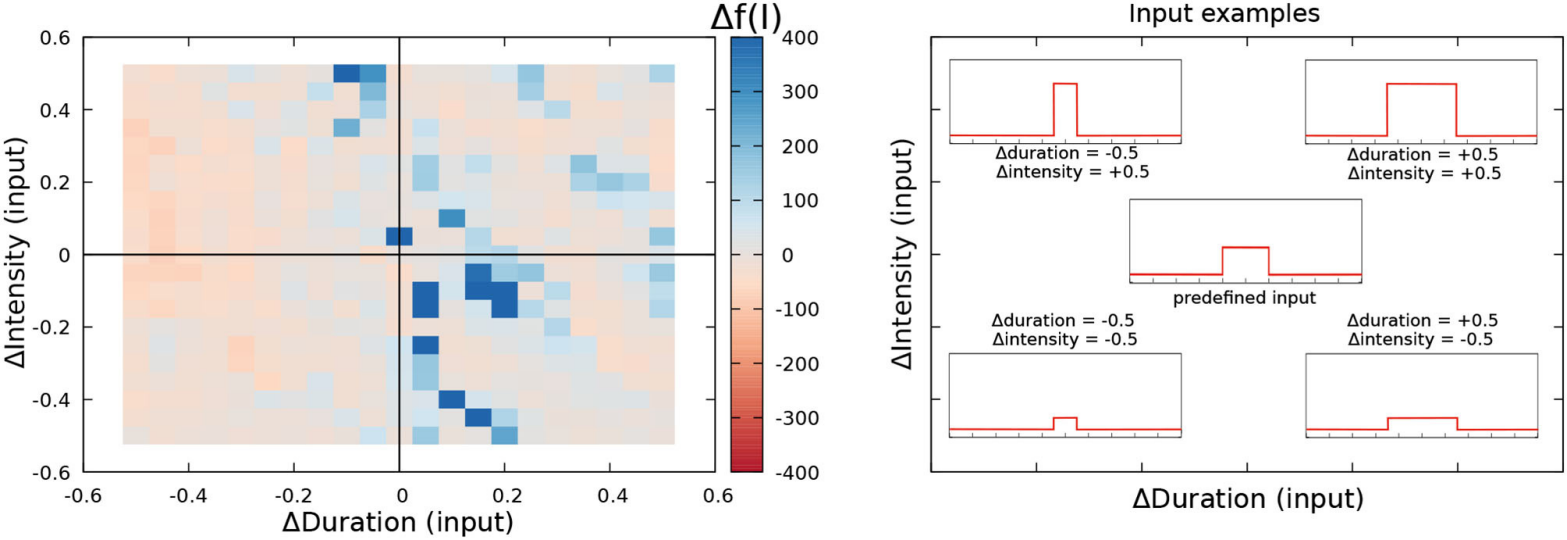
Robustness to input variability analysis of the R-GA electromotor model tuned to recorded *G. petersii* SPI patterns (Left). The central point in the left panel represents the reference fitness value. The one obtained simulating the model under the predetermined simulation conditions (i.e., ΔDuration = 0 and ΔIntensity = 0). Duration and intensity of the step function current input were varied from –0.5 to 0.5 from their initial values, and the relative change in the fitness value was calculated as described by Equation 9. Reddish colors represent decreases in the fitness value under variable stimulation. White colors represent an equivalent result to the reference fitness value. Blueish colors represent an improvement in the fitness value. Representative example of distinct current inputs in the simulation at different places of the chart (Right).

A set of different simulations was conformed by modifying the intensity and duration of the predefined model inputs (i.e., the inputs used in the GA for simulating the patterns shown in Figure 4) up to a 50% to perform this analysis. Being ${\text{In}}_{p}^{0}\left(n\right)$ the predefined value of the input received by nucleus *n* in the *p* pattern simulation case (refer to Supplementary Section 3), and $t_{p}^{0}\left(n\right)$ the duration of the step, the set of simulations to assess robustness is built by modifying intensity from $-0.5\cdot {\text{In}}_{p}^{0}\left(n\right)$ to $0.5\cdot {\text{In}}_{p}^{0}\left(n\right)$ in steps of 0.05, and also modifying duration from $-0.5\cdot t_{p}^{0}\left(n\right)$ to $0.5\cdot t_{p}^{0}\left(n\right)$ using the same interval steps (refer to Figure 5-Right). Then, the reproducibility of the patterns under this set of stimulation cases was evaluated using the fitting function. In this case, a relative fitting value was defined as the change ratio of the fitting value from the default stimulation case. Being *f*^0^ the fitness value of the model under predefined stimulation, and *f*^*i*^ the fitness value under a given stimulation case of the set, then, the relative fitting value (Δ*f*(*I*)) was given by:


(9) $$\begin{array}{l} \Delta f\left(I\right)=\frac{f^{i}-f^{0}}{f^{0}}, \end{array}$$

where negative results mean a decrement in the fitting value when the stimulation inputs vary. When *f*^*I*^ = *f*^0^, Δ*f*(*I*) = 0, which is the case in the central point of robustness charts that we will discuss later (refer to Figure 5-Left).

The robustness of the model is evaluated below in terms of the relative increments and decrements of this value. Strong robustness is defined as the ability to maintain Δ*f*(*I*) > 0. Note that the fitness function was used again to measure the distance to target patterns but no GA was employed.

## 3. Results

A model configuration fitted to reproduce SPI patterns recorded from *G. petersii* specimens (R-GA) was analyzed (refer to Table 1). R-GA reproduced all four SPI patterns with a different level of accuracy and robustness. It is important to note that each specific SPI pattern was evoked only due to the nuclei being stimulated by the inputs (In_*VPd*_, In_*DP*_, In_*PCN*_ in Figure 1), without modifying the model's internal parameters. In regards to the resulting parameter values after GA-optimization, when compared with the initial configuration there was a noticeable increase in the absolute value of the synaptic conductance (*g*_*syn*_) for the ES_PCN_ synapse (an increase of five times R-GA from its initial value). Also, the corollary discharge has a larger maximum release time than any other of the synapses in the model, which is coherent with the idea that it might be an indirect pathway. These characteristics are always present in all best individuals obtained from the GA, and they are also shared with another configuration of the model which was fitted to synthetic patterns using the GA (S-GA, see Supplementary Section 5).

Four different SPIs patterns were simulated in the R-GA model configuration (Figure 4). The resulting scallops in this configuration showed the typical temporal structure associated with this pattern: a sudden drop to short IPIs (around 40 ms) followed by an almost immediate recovery. Also, scallops reached a lower IPI duration than accelerations, in accordance with the activity recorded from the fish.

Accelerations showed a series of almost regular shorter IPIs. IPIs during acceleration were longer (around 85 ms in R-GA), better complying with the accelerations from the fish (which are highly variable, but consistently larger than 20 ms, and even larger in *G. petersii* recordings). It is worth noting that, contrary to what happened in the initial model, in both R-GA and S-GA configurations CN integrated several DP spikes before firing (refer to accelerations in Figure 4, and also Supplementary Figures S5.1, S5.2). Regarding IPIs regularity during the acceleration, IPIs within SPI had approximately the same duration, complying with the regularity in the sequence of short IPIs that define acceleration.

In R-GA configuration, rasps (Figure 4) showed an initial scallop-type decrease to IPIs around 40 ms, followed by a sustained burst of regular short IPIs like in accelerations. Again, in both GA-fitted configurations, CN integrated several DP spikes in their IPIs (refer to rasps in Figure 4 and also Supplementary Figures S5.1, S5.2). Conversely, to what happens with DP, PCN spiking was tightly phase-locked with CN.

Finally, cessations (Figure 4) showed the expected stop in the EOD production during long periods (of around 500 ms).

Results for the robustness analysis to input variability of R-GA are depicted in Figure 5 using a color representation of Δ*f*(*I*) as described in Equation 9. Results did not show relevant decreases in the fitting value for changes up to 50% in the intensity and duration of the default simulation values. Conversely, they showed a limited increase of the fitting value when a slight decrease in duration was balanced with a slight increase in intensity. The result was the same when slight increases in duration were balanced with slight decreases in intensity. These results show that overfitting to simulation timings has been avoided when considering all the patterns at the same time. To also discard overfitting to each SPI pattern, a robustness analysis disaggregated by pattern was also performed (refer to Supplementary Section 10), also showing no relevant drops in the fitness results for any of the patterns. A complementary robustness analysis to test the effect of Gaussian noise on the inputs of the system was also performed (refer to Supplementary Section 9). Equivalent results discarding overfitting were obtained.

**Figure 6:**
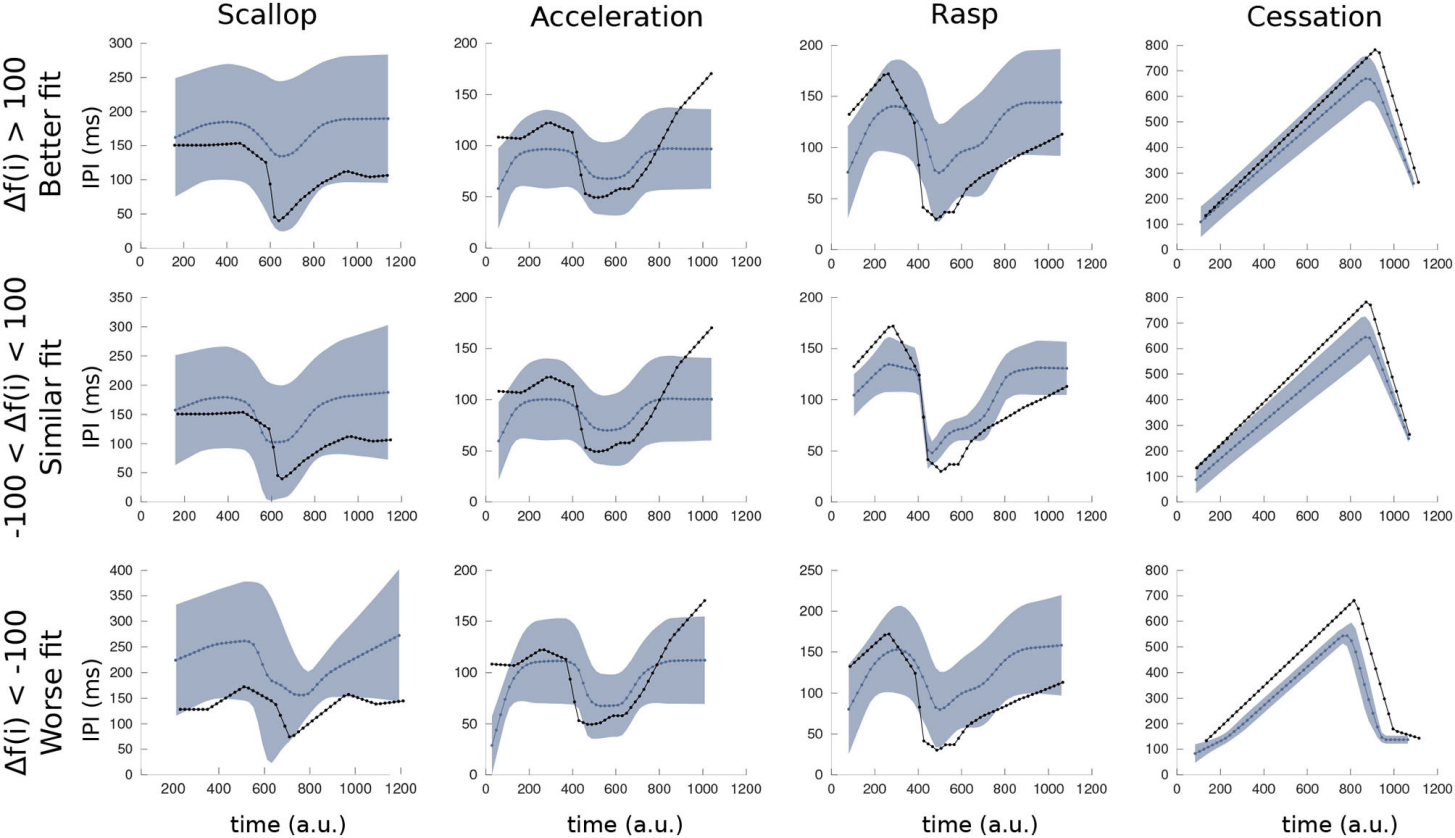
Mean and variance of simulated SPIs using the R-GA model under the variated simulation conditions of the robustness analysis (refer to Figure 5). As in the GA fitness function, the duration of each simulation was normalized to 1,000 arbitrary units for representation, starting at its first IPI. The blue line is the mean SPI from the executions with variated inputs, blue shade represents the SD. Finally, the SPI represented in black is the closest target pattern. Simulations of each SPI pattern were divided into three partitions (with a similar number of elements) according to its fitting value: the ones that obtain better fitting (upper row: Δ*f* < −100), those that obtain similar results to the reference fitness value (center row: −100 < Δ*f* < 100) and the ones that yield worse fitting results (bottom row: 100 < Δ*f*).

In Figure 6, the IPI mean and SD of each SPI pattern simulated during the robustness analysis of the R-GA model are depicted. The line depicted in blue is the mean SPI of the several executions with variable inputs, and the shade represents the SD of the results. Finally, the black line corresponds to the closer target pattern used to calculate the fitness. The simulation of each SPI pattern was divided into three partitions according to its fitting value: Worse fit than the one obtained with default inputs (Δ*f* < −100); similar fit (−100 < Δ*f* < 100); and better fit (Δ*f* > 100). It is shown that even in the worst fit results, the internal temporal characteristic of each SPI pattern was reproduced: the sudden drop to the shortest IPI followed by an almost immediate recovery in the scallop; a series of almost regular short IPIs (above the minimum IPI in scallops) in accelerations; a sudden drop followed by an acceleration-like tail of regular IPIs; and a long pause of hundreds of milliseconds in EOD production during cessations. Rasps were the least robust SPI pattern in R-GA, but even those with the worsts fitting results kept a recognizable rasp shape.

**Figure 7:**
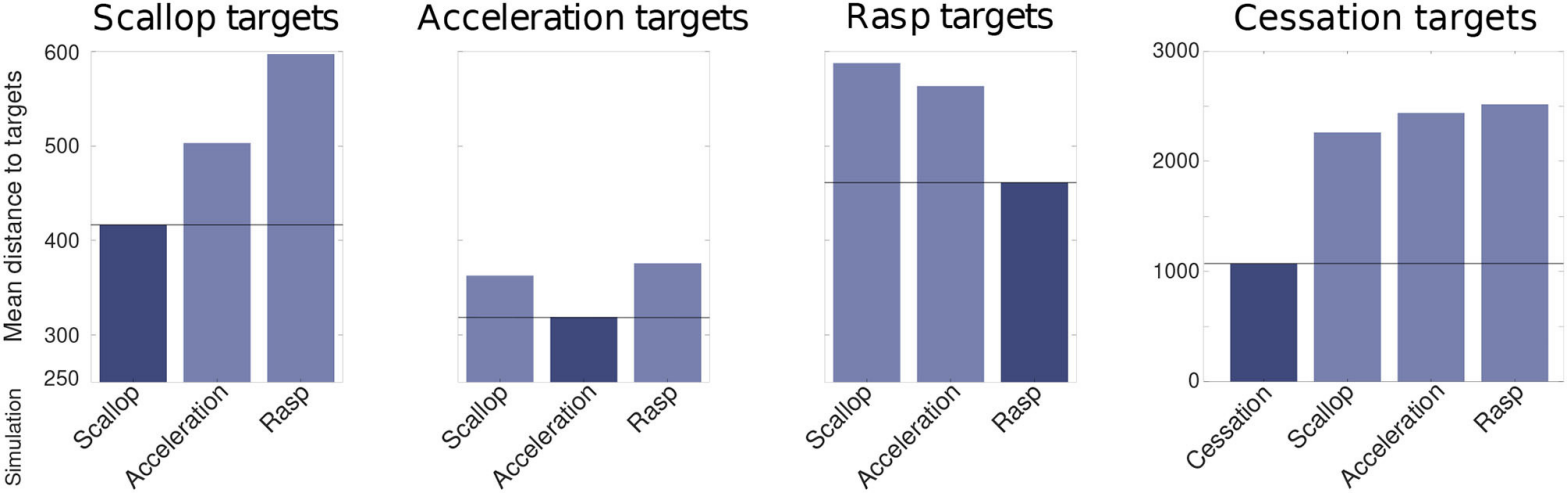
Mean euclidean distance (calculated after the evaluation function steps depicted in Figure 3) between each target SPI pattern and the simulated SPIs during the R-GA robustness analysis. Each chart shows the distance calculated to a different target SPI: scallop, acceleration, rasp, and cessation; and the darkest bar in each chart highlights the simulations corresponding to that target. This bar was expected to be minimum in each case as it represents the mean distance between the set of simulations of a specific SPI and its corresponding target. This held true for all cases. Differences from cessation simulations are not depicted to improve the clarity of the comparison because they are in a larger order of magnitude.

The robustness analysis also showed that the differences between simulations of distinct SPIs were larger than the differences between simulations of the same SPI with variated inputs (refer to Figure 7). To measure this difference, the mean Euclidean distance between each target SPI pattern and the simulated SPIs during the R-GA robustness analysis was calculated. Each chart in Figure 7 shows the distance calculated to a different target SPI, and the darkest bar in each chart highlights the simulations corresponding to that target. This bar was expected to be minimum in each case as it represents the mean distance between the set of simulations of a specific SPI and its corresponding target. This held true for all cases. Thus, for all simulations during the robustness analysis, the differences between distinct SPIs were larger than the differences between simulations of the same SPI obtained from variated inputs.

## 4. Discussion and Conclusion

Computational models have been used to answer different questions in the study of electroreception and electrogenesis. Regarding electrogenesis, Moortgat et al. (2000), Shifman et al. (2015), and Lucas et al. (2019) used an anatomically detailed model of the pacemaker of *Apteronotus leptorhynchus* to study the electric organ signal and its spatiotemporal features in wave-type fish. In Shifman and Lewis (2018) a model for the *Eigenmannia* was employed to address the jamming avoidance response in these fish. Electroreceptor models are of particular interest as they have a relevant role in understanding the electrical sense, even in non-electroactive species. On the other hand, bioinspired approaches have built robotic electrosensory systems at different abstraction levels to mimic electrolocation. In this context, a robotic model of electric fish fins was developed to engage in electrocommunication with living fish, study fish maneuverability and develop an underwater autonomous robot with electric sense (Neveln et al., 2013; von der Emde and Worm, 2021).

Despite the variety of models regarding signal generation in weakly electric fish, the development of a computational model of the electromotor command network that can produce the variability of SPIs observed in pulse mormyrids had not been attempted until now.

The modeling study presented here assesses the relevance of different parameters in the electromotor command network for reproducing as a whole the diverse temporal structure of output patterns displayed by the living system. Our results suggest that the diversity of SPIs shown by the system results from a dynamic balance of intensity and timing between the synaptic properties of the network. This highlighted the hypothesis that relevant synaptic parameters (and not only the nucleus dynamics or the network topology) play an important role in reproducing the whole set of SPIs observed in experimental data.

A multi-objective genetic algorithm (GA) was developed and applied to tune the synaptic parameters of the model. The proposed GA optimization method allowed us to readily obtain different parameter configurations, which optimize the ability of the model to reproduce all target patterns under different activations in the electromotor command network.

Automatic synaptic parameter optimization allowed to prove that it is possible to reproduce the four pre-selected SPI patterns using the same intrinsic dynamics and connectivity reaching a dynamic balance between the synaptic properties in the network. Supplementary Material provides complementary analysis examining different topologies (Supplementary Sections 6, 7), nuclei dynamics (Supplementary Section 8), and synaptic inputs (Supplementary Section 9) supporting this conclusion.

In the resulting model each nucleus is represented by a single (Izhikevich, 2003) unit because of the lack of biological details below the nucleus level, neither on the constituent neurons nor on the internal connectivity. The optimization process on synaptic parameters is key in this regard to deal with this lack of detail. Connections in this model integrate synapses that occur between neurons in different nuclei in the real network. This reinforces the importance of synaptic dynamics above other parameters in the model to robustly generate the variability of observed patterns.

It was observed during the fitting process that the different configurations of the model are sensitive to even slight changes in synaptic parameters, resulting in significant drops in the model performance, both in terms of the fitting value and the shape of the simulated SPI patterns (for more details about the continuity in the parameter space between the different configurations, refer to Supplementary Section 12). This does not mean that configurations are unique, as different configurations could lead to similar results, but that they must show a delicate balance between synaptic intensities and timings to succeed in reproducing the SPI patterns.

Regarding the changes in the simulation input parameters (duration or intensity of the stimulus presented to the model), sometimes they also lead to losses of SPIs internal structure but, as long as the proper nucleus/nuclei is/are stimulated, it does not occur in such a critical way as it happens with changes in the synaptic parameters (refer to Supplementary Section 11). Robustness results show that overfitting to the stimulation inputs was avoided. Although there are also drops in fitness for certain combinations of inputs, the general tendency was to maintain (and sometimes even improve) the performance of the model under different stimulation (as shown in Figure 5). These robustness results point out that SPI generation depends on the nucleus activation and not on the intensity or the duration of the inputs.

The implemented model and its computational efficiency also enable novel closer-to-natural stimulation techniques to perform more realistic closed-loop experiments with the real system (as pulse-type mormyrids have been previously used in several closed-loop studies where stimulation is guided by the fish's own activity, as in Forlim and Pinto, 2014; Forlim et al., 2015; Lareo et al., 2016). In this context, we can expect these experiments to benefit from a more realistic stimulator based on the described model (Lareo et al., 2016). The model also allows further studies of the underlying mechanisms of electrocommunication, including its use in robotic fish. Nevertheless, the internal relationship between fish skin electroreceptors and the electromotor system is yet to be further studied.

Beyond the mormyrids electromotor command network, many other neural systems also produce outputs with characteristic stereotyped temporal structures. However, it is not an easy task to design a multiobjective modeling approach flexible enough to automatically tune a model to several temporal structured targets. The methodology described here, particularly the fitness function and the GA, can be adapted to fine tune the model of other neural networks also exhibiting a whole set of sequential output patterns.

All the software developed and used in the present analysis (the model, the multi-objective GA used for parameter adjustment, the fitness function to represent and compare different sequences of temporal firing patterns, and the robustness analysis software) is made available^1^ for future studies of this network and similar networks in other animals (refer to Supplementary Section 13). We expect this open-source contribution to help deepen our understanding of the role of ensemble connectivity and the synaptic mechanisms shaping the functional patterns of neural temporal structures.

## Data Availability Statement

The raw data supporting the conclusions of this article will be made available by the authors, without undue reservation.

## Ethics Statement

The animal study was reviewed and approved by Ethics Committee of Universidad Autónoma de Madrid (TIN2017-84452-R/CEI-88-1661).

## Author Contributions

The model was conceived and designed by AL and FR. Literature review was done by AL, FR, and PV. AL performed the experimental recordings and runs both the simulations and the GA optimization. AL and FR analyzed the results. AL, FR, and PV wrote the manuscript. All authors contributed to the article and approved the submitted version.

## Funding

This study was supported by AEI/FEDER grants TIN2017-84452-R, PID2020-114867RB-I00, and PGC2018-095895-B-I00. The funders had no role in study design, data collection, analysis, decision to publish, or manuscript preparation.

## Conflict of Interest

The authors declare that the research was conducted in the absence of any commercial or financial relationships that could be construed as a potential conflict of interest.

## Publisher's Note

All claims expressed in this article are solely those of the authors and do not necessarily represent those of their affiliated organizations, or those of the publisher, the editors and the reviewers. Any product that may be evaluated in this article, or claim that may be made by its manufacturer, is not guaranteed or endorsed by the publisher.

## Footnotes

^1^Code is available in the following github repository: https://github.com/GNB-UAM/electromotor-nmodel/.
