# Supplementary material for: Modeling the Sequential Pattern Variability of the Electromotor Command System of Pulse Electric Fish
Source: Front Neuroinform. 2022 Jun 28;16:912654. doi: 10.3389/fninf.2022.912654 (PMC9275807; doi:10.3389/fninf.2022.912654)
Supplement: Supplementary file 1 [file Data_Sheet_1.pdf]

Supplementary Material

1 TARGET SPI PATTERNS: SYNTHETIC AND RECORDED

Synthetic SPIs were *ad-hoc* defined based on the characteristic temporal structure of each SPI pattern, as described by previous studies in different species of mormyrids (Moller et al., 1989; Carlson and Hopkins, 2004; Caputi et al., 2005). Different examples of synthetic SPIs were generated for each behavioral pattern (scallop, acceleration, rasp, and cessation). Synthetic data was used for S-GA tuning and for detecting SPI patterns in *Gnathonemus petersii* recordings, as described below. Bottom panels of Fig 2 in the paper show just one example set of these synthetic patterns. The timing characteristics of each SPI pattern are precisely described in paper section 2.1 *Characteristic sequences of pulse interval (SPIs)*, and are briefly repeated here for clarity:

- Accelerations are sustained IPI shortenings to a series of almost regular shorter IPIs.
- Scallops are sudden drops to very short IPIs (its minimum IPI use to be below the minimum IPI in accelerations) followed by an almost immediate recovery to regular resting IPIs.
- Rasps are a type of IPI pattern that has an initial sudden decrease to very short IPIs, similar to the one observed in scallops, followed by a step increase of IPIs duration for a sustained series of IPIs, like a long tail of short regular IPIs similar to the ones observed in the acceleration pattern.
- Cessations correspond to activity dropping in the EOD generation for periods up to one second.

Recorded SPIs (used in R-GA optimization) were obtained from three different freely-behaving *Gnathonemus petersii* specimens in an 80 l water tank, using four differential dipoles placed in the tank forming 45º angles between them (see Lareo et al. (2016) for more details). The grounding electrode was also located in the aquarium. The signal from the dipoles was amplified (TL082 JFET-Input Dual Operational Amplifier; gain ≈ 45x), summed (LM741 Operational Amplifiers), squared (using AD633 Analog Multiplier), and then digitized at 15 kHz by a DAQ board (NI PCI-6251, National Instruments Corporation, 16 bit Analog resolution).

IPIs detection was performed from continuous time series data using a crossing threshold algorithm for spike detection, as described in more detail in Lareo et al. (2016). Pulse-intervals are calculated subtracting from each spike time the time from the preceding spike. One example set of SPIs recorded from *Gnathonemus petersii* specimens are depicted in top panel of paper Fig 2. To facilitate and automate the SPI search in the *Gnathonemus petersii* recordings, sequences of pulse intervals were identified using the fitness function (described in section 2.4.1 of the paper) by evaluating them against the synthetic SPI targets. Thus, the SPIs from the recordings that best matched the temporal structure of each target pattern were selected. As described in section 2.4.1 of the paper, during evaluation, SPIs were normalized to the same duration (1000 arbitrary units) and linearly interpolated (every 20 arbitrary units, for a total of $n = 50$ points for each pattern) to characterize the pattern shape.

The complete dataset of synthetic and recorded SPI patterns applied for GA optimization is provided with the source code[1].

[1] See yaml configuration files in the Github repository: https://github.com/GNB-UAM/electromotor-nmodel/configs

Permission from the ethics committee of Universidad Autónoma de Madrid was obtained (TIN2017-84452-R/CEI-88-1661). All experiments were noninvasive behavioral trials. All animals behaved normally after the experiments.

2 MODEL PARAMETERS

The electromotor system was modeled using four nuclei and five synapses. The nuclei VPd, DP, PCN, and CN, were simulated using the neuron model developed by Izhikevich et al. (2003), where a wide range of neuron dynamics can be evoked by selecting different values of the parameters a, b, c and d (Destexhe et al., 1994). These values were tuned ad hoc to reproduce dynamics described by previous neurophysiological studies. The following firing regimes were used:

- Low-threshold spiking (LTS): A type of low firing threshold activity typically observed in inhibitory cortical cells. In this mode, a neuron can fire high-frequency trains of spikes (burst), and it also shows frequency adaptation while the stimulation is sustained (see table S.2). The parameters of VPd in the model correspond to this kind of behavior.
- Regular frequency spiking (RFS): This mode corresponds to spike firing at a regular frequency when stimulation amplitude is held constant (see table S.2). It is the firing regime in which DP operates.
- Regular Spiking (RS): This mode increases its regular firing frequency when stimulated (see table S.2). Adaptation to the stimulus results in recovery from short interspike intervals to the regular firing frequency. PCN follows this behavior.
- Phasic Spiking (PS): This firing regime operates as an integrate-and-fire model, although it fires only a single spike as a response to the input and remains quiescent afterward (see table S.2). In the model, it is the firing regime used for CN.

Table S.1 shows the parameter values used in the electromotor model. Figures in table S.2 show the activity of the individual dynamics of all nucleus types considered in the model, and its response to input synaptic stimulation from the network model.

The synapses (IS_{DP}, IS_{PCN}, ES_{DP}, ES_{PCN}, and ES_{CDP}) were simulated using the model described by (Destexhe et al., 1994). The most relevant parameters of the model (α, β, gsyn and t_{max}) were automatically adjusted to the target SPI patterns using the genetic algorithm (GA) described in this paper. Table 3 shows the initial ad hoc tuned configuration (S-T).

S.1 Supplementary Table. Neuron parameters of the model to reproduce the dynamics of the electromotor nuclei as described by previous neurophysiological studies (Caputi et al., 2005) (see Eq. 1, Eq. 2 and Eq. 3).

Nucleus	Dynamics	a	b	c	d
VPd	Low-threshold spiking (LTS)	0.02	0.25	-65	2
DP	Regular frequency spiking (RFS)	0.1	0.26	-65	2
PCN	Regular spiking (RS)	0.02	0.2	-65	8
CN	Phasic spiking (PS)	0.02	0.25	-65	6

3 SIMULATION PARAMETERS

To simulate the electromotor command system, VPd, DP, and PCN received external currents, representing inputs. These predefined values represent current inputs received by the nuclei from other sources of the

S.2 Supplementary Table. Dynamic properties of the model nuclei chosen in this study to match their biological counterparts. Values for the parameters of the model by (Izhikevich et al., 2003) were tuned to replicate the desired experimental dynamic (parameter values are detailed in supplementary section 2). The last column shows the activity of each isolated nucleus model in response to an input step current and to synaptic stimulation ($I_{syn}(t)$ in Eq. 1) from the model (black line at the bottom represents the time during which the network is being stimulated).

<table>
<tr><th>Nucleus</th><th>Desired dynamic</th><th>Nucleus model response</th></tr>
<tr><td>VPd</td><td>Low threshold spiking</td><td>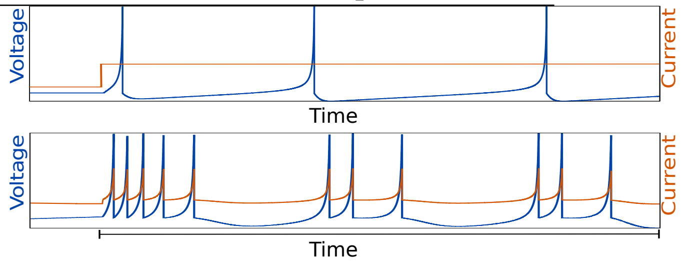
</td></tr>
<tr><td>PCN</td><td>Frequency adaptation</td><td>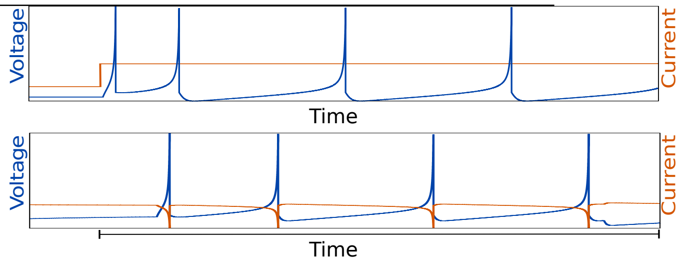
</td></tr>
<tr><td>DP</td><td>Regular spiking</td><td>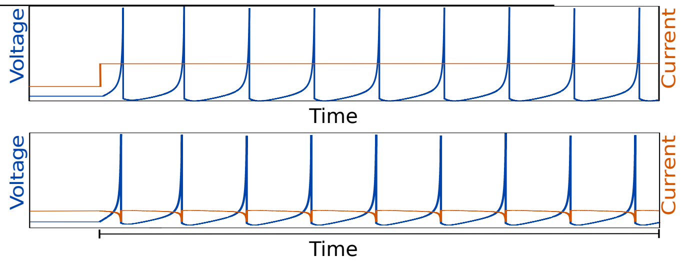
</td></tr>
<tr><td>CN</td><td>Integrate and fire</td><td>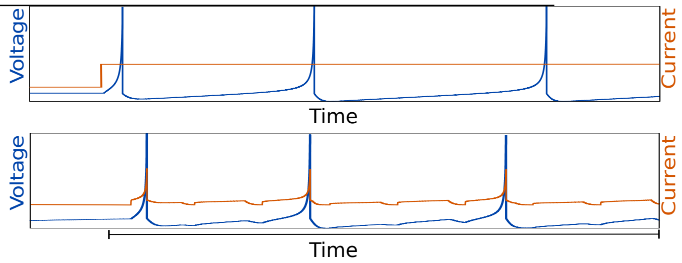
</td></tr>
</table>

nervous system. The following tables show the current input values for simulating scallops (S.4 Table), accelerations (S.5 Table), rasps (S.6 Table) and cessations (S.7 Table). The first row in each table represents simulation time (in milliseconds), and subsequent rows represents neuron current input for each nucleus model (VPd, DP, PCN) during this time. These inputs were step-functions used to simulate the SPIs shown in Fig. 4. Also, these are the predefined simulation parameters for the GA evolution and the $\mathrm{In}_p^0(n)$ robustness analysis (center values in Fig. 5 and Fig. 10.1).

4 GA OPTIMIZATION

Each GA result is based on a minimum of 30 GA runs with different GA parameters (changes in parameter space limits and GA stop conditions, see Table 8), which were launched concurrently. S-GA and R-GA

S.3 Supplementary Table. Relevant synapse parameters of the *ad hoc* tuning to synthetic patterns (S-T) and the GA-fitted to synthetic patterns (S-GA) configuration of the model (*threshold* = 0; $E_{syn} = -80$; T = 1; see Eq. 4 and Eq. 5) and the .

		**S-GA**		
Synapse	α	β	g_{syn}	t_{max}
IS_{DP}	9.056	2.722e-3	-1.251e-1	223.097
IS_{PCN}	7.501	2.710e-2	-2.763e-1	169.763
ES_{DP}	4.949	1.273e-1	1.795e-1	78.9001
ES_{PCN}	4.949	1.485e-1	2.595e-1	78.9001
ES_{CDP}	4.795	3.274e-3	7.056e-1	396.811

		**S-T**		
Synapse	α	β	g_{syn}	t_{max}
IS_{DP}	5	0.005	-0.12	160
IS_{DP}	5	0.005	-0.15	160
ES_{DP}	5	0.1	0.1	30
ES_{DP}	5	0.18	0.05	30
ES_{DP}	5	0.02	0.3	400

S.4 Supplementary Table. Scallop simulation parameters. This SPI pattern is evoked by increasing current input to PCN nucleus. VPd input (-0.5 mA) and DP input (1.7 mA) are constant in this pattern.

Time (ms)	[0, 520]	[520, 680]	[680, 1200]
PCN input	6.5	14	6.5

S.5 Supplementary Table. Acceleration simulation parameters. This SPI pattern is evoked by increasing current input to DP nucleus. VPd input (-0.5 mA) and PCN input (6.5 mA) are constant in this pattern.

Time (ms)	[0, 800]	[800, 1200]	[1200, 2000]
DP input	1.7	4	1.7

S.6 Supplementary Table. Rasp simulation parameters. This SPI pattern is evoked by increasing current input to both DP and PCN nuclei. VPd input (-0.5 mA) is constant in this pattern.

Time (ms)	[0, 500]	[500, 580]	[580, 830]	[830, 1330]
DP input	1.7	4.5	4	1.7
PCN input	6.5	15	7	6.5

models were obtained after several iterations of these runs. At least 4 iterations (and therefore 120 total GA executions) for both S-GA and R-GA were performed.

A steady-state GA scheme [2] was used. In this kind of GA, the overlap between generations results in faster convergence where, once an individual improves its fitting value, all the individuals reach similar fitting value in just a few generations. As a result, all the generations in the GA tended to have a mean

[2] http://lancet.mit.edu/galib-2.4/API.html

S.7 Supplementary Table. Cessation simulation parameters. This SPI pattern is evoked by increasing current input to VPd neuron. DP input (1.7 mA) and PCN input (6.5 mA) are constant in this pattern.

Time (ms)	[0, 250]	[250, 650]	[650, 1000]
VPd input	-0.5	8	-0.5

fitting value near the maximum, resulting in low parameter variance (see Table S.9). This GA scheme was the one that gave better results in Lareo et al. (2018).

Regarding convergence, most runs ended improving the initial fitting behavior and without increases during at least the last 100 generations. Convergence time ranged from 3 to 8 days using an HPC cluster with different kinds of processors: AMD Opteron(tm) Processor 6344, AMD EPYC 7451 24-Core Processor and Six-Core AMD Opteron(tm) Processor 2435. The software provided is single-threaded so the advantage of using a cluster come from running multiple simulations (a parallel version of the GA optimization is being developed), so the main differences in the convergence time are due to different stopping criteria between executions: number of generations or relative increase of the initial fit (see Stopping criteria in Supplementary Table S.8). Therefore, for instance, the minimum convergence time (3 days) corresponds to 400 generations running in the EPYC 7451; the maximum convergence time (8 days) corresponds to a relative increase of 2 in the initial fit running in the Opteron 2435. Also, see results from previous versions of the GA in Lareo et al. (2018). The possibility of configurations being stuck in a local minimum can never be totally discarded, although we consider this hypothesis unlikely given that the percentage of variation considered (50 to 120 percentual variation from the initial values), that different GA schemes were previously tested, and that 120 executions of the GA were performed.

S.8 Supplementary Table. Set of GA execution parameters used in 30 different runs of the GA. Each run in the set was performed at least 4 times (i.e., at least 120 runs of the GA). When defining the valid parameter space as a range, the mean value of the range for a parameter is given by its initial value. The minimum value for each parameter is 0. When using relative increases as the stopping criteria, GA runs were also limited to 5000 generations.

Stopping criteria		Parameter space	
Number of generations	400	Percentual variation from initial value	50%
	800		50%
	1600		50%
	400		90%
	800		90%
	1600		90%
	400		120%
	800		120%
	1600		120%
	400	Range variation from initial value	$\alpha \pm 2.5$; $\beta \pm 0.05$; $g_{syn} \pm 0.45$; $t_{max} \pm 70$
	800		$\alpha \pm 2.5$; $\beta \pm 0.05$; $g_{syn} \pm 0.45$; $t_{max} \pm 70$
	1600		$\alpha \pm 2.5$; $\beta \pm 0.05$; $g_{syn} \pm 0.45$; $t_{max} \pm 70$
	400		$\alpha \pm 4.5$; $\beta \pm 0.05$; $g_{syn} \pm 0.45$; $t_{max} \pm 70$
	800		$\alpha \pm 4.5$; $\beta \pm 0.05$; $g_{syn} \pm 0.45$; $t_{max} \pm 70$
	1600		$\alpha \pm 4.5$; $\beta \pm 0.05$; $g_{syn} \pm 0.45$; $t_{max} \pm 70$
Relative increase of initial fit	0.5	Percentual variation from initial value	50%
	1		50%
	2		50%
	0.5		90%
	1		90%
	2		90%
	0.5		120%
	1		120%
	2		120%
	0.5	Range variation from initial value	$\alpha \pm 4.5$; $\beta \pm 0.05$; $g_{syn} \pm 0.45$; $t_{max} \pm 70$
	1		$\alpha \pm 4.5$; $\beta \pm 0.05$; $g_{syn} \pm 0.45$; $t_{max} \pm 70$
	2		$\alpha \pm 4.5$; $\beta \pm 0.05$; $g_{syn} \pm 0.45$; $t_{max} \pm 70$
	0.5		$\alpha \pm 4.5$; $\beta \pm 0.05$; $g_{syn} \pm 0.45$; $t_{max} \pm 70$
	1		$\alpha \pm 4.5$; $\beta \pm 0.05$; $g_{syn} \pm 0.45$; $t_{max} \pm 70$
	2		$\alpha \pm 4.5$; $\beta \pm 0.05$; $g_{syn} \pm 0.45$; $t_{max} \pm 70$

S.9 Supplementary Table. Mean and standard deviation of the relative fitting values from last generation of the GA execution used to obtain each configuration.

	SGA	RGA
μ	$-1.12 \cdot 10^{-2}$	$-4.05 \cdot 10^{-2}$
σ	$1.38 \cdot 10^{-6}$	$3.05 \cdot 10^{-5}$

5 S-T AND S-GA CONFIGURATIONS OF THE MODEL

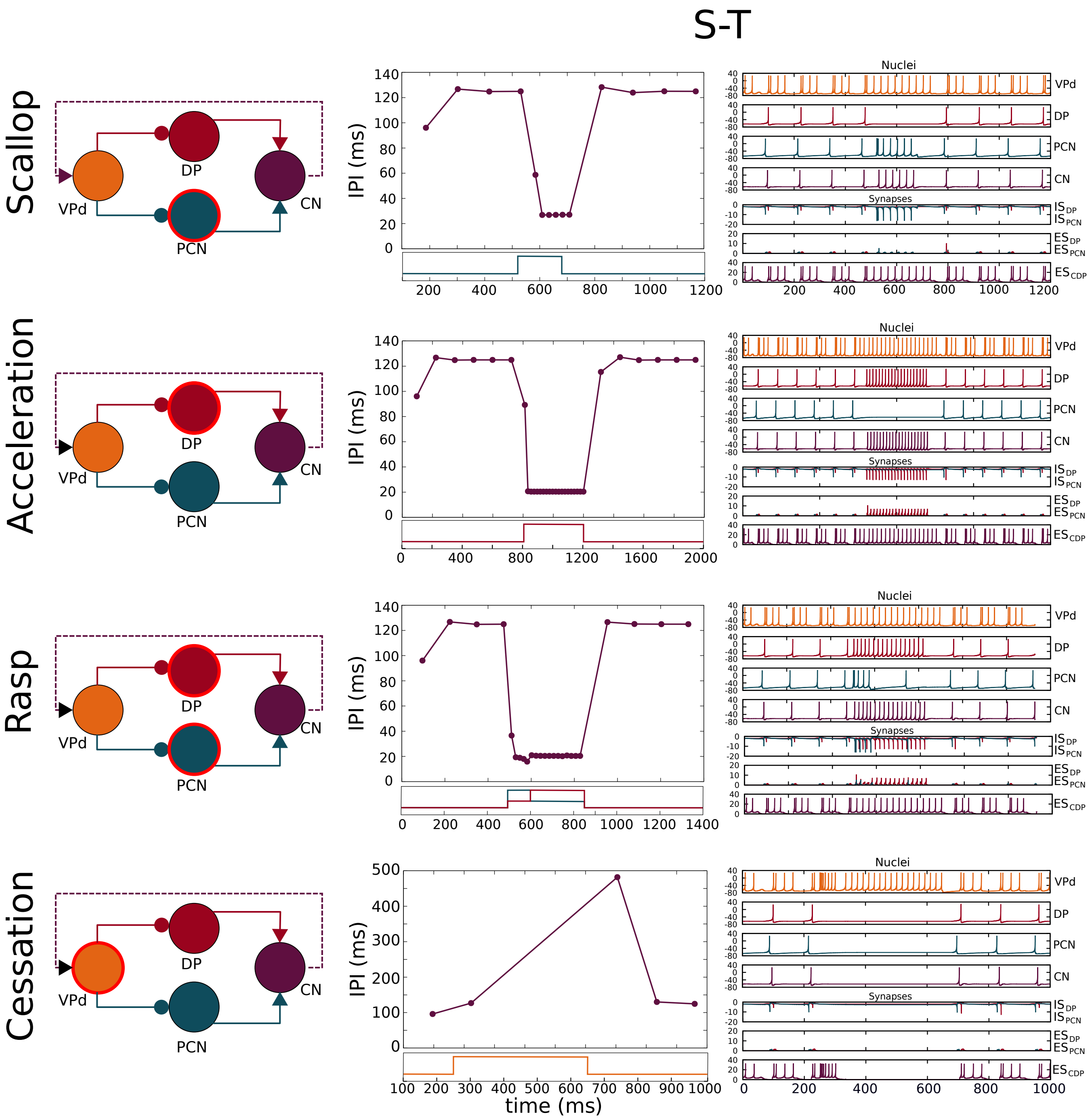

Supplementary Figure 5.1: Simulation of the four SPI patterns in the *ad hoc* tuned configuration (S-T). Each row showed (left) a schematic of the network where the nucleus/nuclei responsible for generating the SPI (Caputi et al., 2005) is/are highlighted using a circular red stroke. The results are depicted in two columns: the first one shows the SPIs resulting from the simulation, and the second one shows the nuclei voltages and synaptic currents. Each chart is related to its corresponding nuclei/synapse by color. Step functions used as current inputs to activate nuclei/nucleus are depicted under each SPI pattern, and they are also related to their corresponding nuclei by color. The model parameters are described in Table S.3. Simulation parameters used in the simulations are described in supplementary section 3.

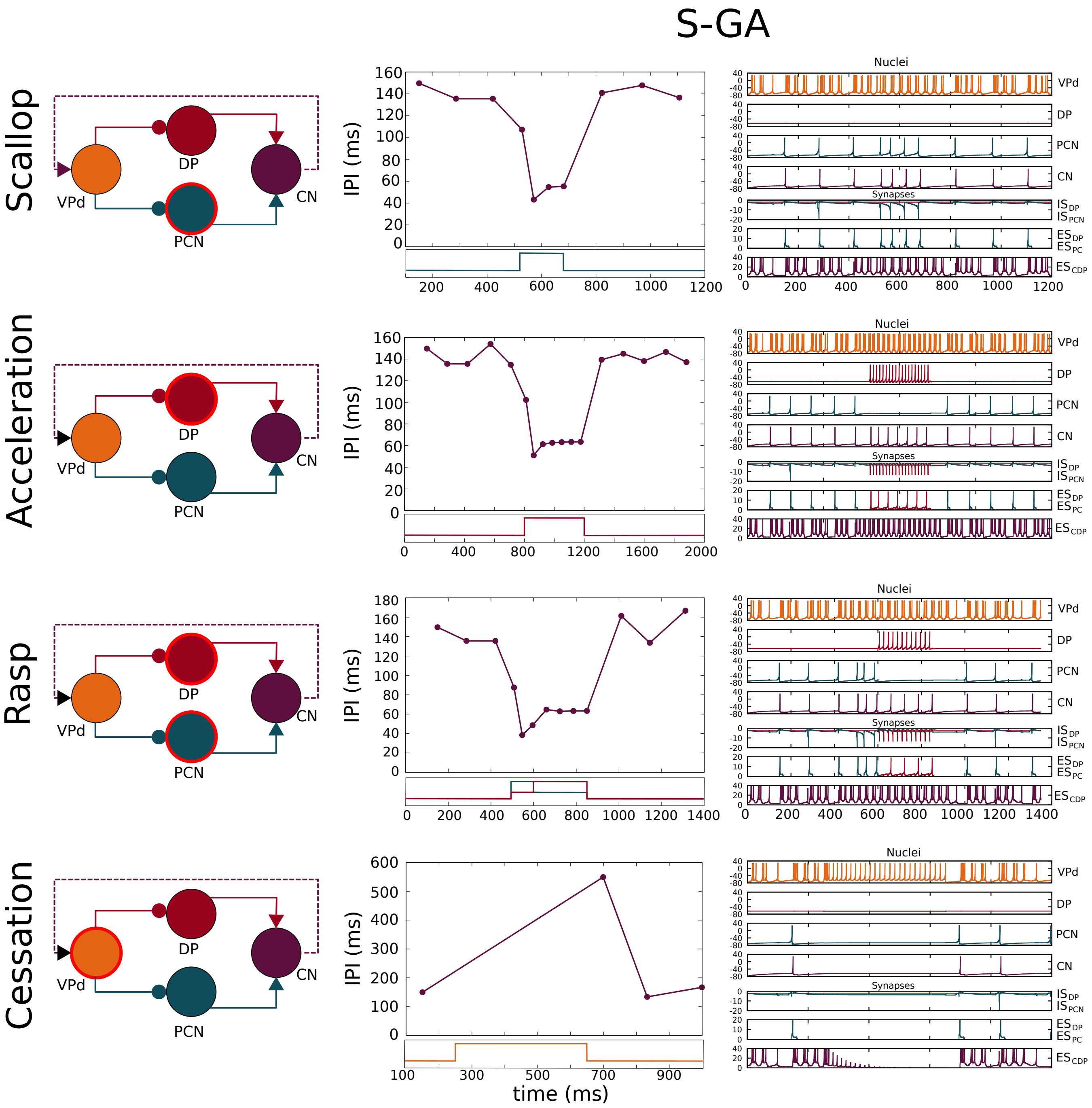

Supplementary Figure 5.2: Simulation of the four SPI patterns in the model configuration optimized to synthetic examples (S-GA). Each row showed (left) a schematic of the network where the nucleus/nuclei responsible for generating the SPI (Caputi et al., 2005) is/are highlighted using a circular red stroke. The results are depicted in two columns: the first one shows the SPIs resulting from the simulation, and the second one shows the nuclei voltages and synaptic currents. Each chart is related to its corresponding nuclei/synapse by color. Step functions used as current inputs to activate nuclei/nucleus are depicted under each SPI pattern, and they are also related to their corresponding nuclei by color. The model parameters are described in Table S.3. Simulation parameters used in the simulations are described in supplementary section 3.

Four different SPIs patterns were simulated in R-GA (Fig. 5.1 and 5.2). Scallop simulations in both S-GA and R-GA showed the typical temporality associated with this pattern (a sudden drop to short IPIs (around 40 ms) followed by an almost immediate recovery). The shorter IPI reached in S-T was slightly faster ($\sim$25 ms), although the burst duration in all three configurations remained almost the same. Both in S-GA and R-GA, scallop SPIs reached lower IPI duration than accelerations, in accordance with the activity recorded from the fish.

In all the three configurations, accelerations (Fig. 4 - B) showed a series of almost regular shorter IPIs. In S-T, short IPIs during acceleration was around 20 ms. In S-GA, IPIs during acceleration were longer (around 60 ms. in S-GA and 85 ms in R-GA), better complying with the accelerations from the fish (which are highly variable, but consistently larger than 20 ms, and even larger in *Gnathonemus petersii* recordings). It is worth noting that, contrary to what happened in the initial S-T model, in both GA-fitted configurations, CN integrated several DP spikes before firing. Regarding IPIs regularity during the acceleration, IPIs within SPI had approximately the same duration. Both R-GA and S-GA configurations showed regularity in the sequence of short IPIs that define acceleration, although in S-GA, the starting IPI tended to be slightly shorter.

Regarding S-T results, scallop and rasp SPI patterns cannot be easily distinguished, as both showed acceleration-like short regular IPIs (Fig. 4). The total SPI duration was different for each pattern, but S-T rasps and scallops lacked their characteristic internal structure.

In S-GA and R-GA, rasps (Fig. 4 - C) showed an initial scallop-type decrease to IPIs around 40 ms, followed by a sustained burst of regular short IPIs like in accelerations. As mentioned before, in S-T, the pattern was not fully identifiable. In S-GA, the IPIs were shorter, so a larger number of IPIs formed the SPI. Also, the recovery to larger IPIs was more abrupt in this case. Again, in both GA-fitted configurations, CN integrated several DP spikes in their IPIs. Conversely, PCN spiking was tightly phase-locked with CN.

Finally, cessations (Fig. 4 - D) showed the expected stop in the generation of pulses during long periods (of around 500 ms) in all three configurations.

Regarding the parameter results after GA-optimization, GA configurations showed some shared characteristics between them when compared with the S-T configuration. It was the increase in the absolute value of the synaptic conductance (g_{syn}). Note that some of these parameters underwent noticeable changes from its initial value in S-T. This is the case of the g_{syn} parameter for the ES_{PCN} synapses (an increase of four times for S-GA and five times R-GA from its initial value in S-T, as it can be seen in Table 1 in the paper and Supplementary Table S.3). These similarities are remarkable as network parameters are fitted to a different set of samples of the same SPI patterns. Also remarkable is that S-GA showed similar α, β and t_{max} parameters for both ES_{DP} and ES_{PCN} projections (Table S.3), which means that the timing of the synapses is almost symmetrical for both excitatory pathways (Fig. 1).

Analysis of the different GA-fitted configurations of the model (described in Table 1 in the paper and Table S.3) can be summarized in the following observations:

- The temporal characteristics of the corollary discharge pathway are shared between configurations. In they all, the corollary discharge have a larger maximum release time than any other of the synapses in the model. This characteristic is always present in all best individuals obtained from the GA and is coherent with the idea that it might be an indirect pathway.
- S-GA showing similar α, β, and t_{max} parameters for both ES_{DP} and ES_{PCN} projections (see Table 1 in the paper) reinforces the hypothesis of synaptic intensity being the decisive parameter in the elicitation

of accelerations or scallops. Nevertheless, the conductance ratio between these synapses is not the same in R-GA. It denotes that the relation between synaptic strengths determines the output of the system in a non-linear manner.

- The model is sensitive to changes in the synaptic parameters for reproducing all SPIs. Even slight changes on them sometimes result in severe performance drops (see supplementary section 11).

6 HOMOGENEOUS PHASIC SPIKING NEURONS

In order to address the contribution of the ad-hoc tuned nuclei parameters to the overall network output, all nuclei (VPd, DP, PCN, CN) were adjusted to homogeneous parameters reproducing a phasic spiking behavior. GA was then applied to fit synaptical parameters to both recorded and synthetic data. Fitting results (calculated using the fitness function) were consistently lower than those obtained in S-GA and R-GA. The best result obtained is depicted in Fig. 6.1. In this case, all SPI patterns were still recognizable. This denotes that the nuclei's intrinsic characteristics do not directly determine SPIs temporal structure. On the contrary, this reinforces the idea that synaptic conductances in the mormyrids electromotor system play a primary role in the generation of the temporal structure of SPI patterns.

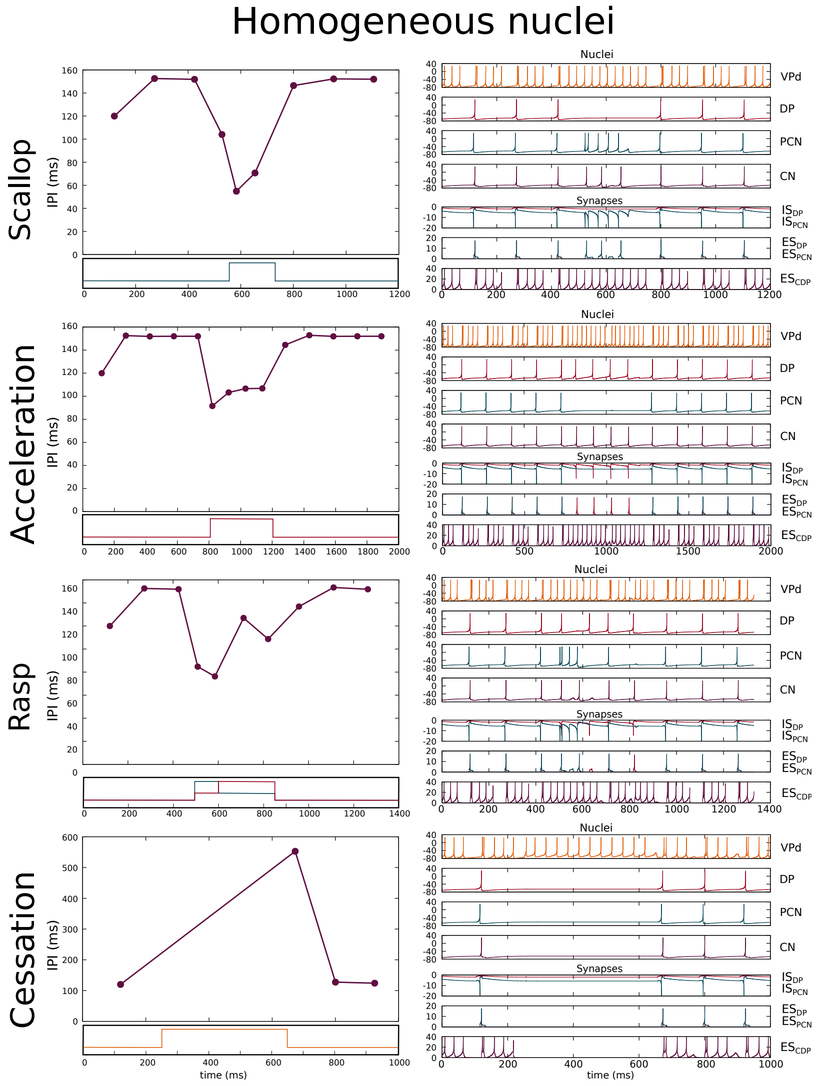

Supplementary Figure 6.1: Simulation of the four SPI patterns in the best fitting model using homogeneous nuclei parameters reproducing a phasic spiking behavior. The results are depicted in two columns: the first one (left) shows SPIs resulting from the simulations and the second one (right) shows the nuclei voltages (top) and synaptic currents (bottom).

7 NO COROLLARY DISCHARGE

To address how a corollary discharge pathway (CDP) contributes to the variability of SPI patterns showed by the mormyrids electromotor system, CDP synapse (ES_{PCN}) was removed from the original model. GA was then applied to fit synaptical parameters (for the rest of synapses) to both recorded and synthetic data. Fitting results calculated using the fitness function were consistently lower than those obtained in S-GA and R-GA. The best result obtained in our tests is depicted in Fig. 7.1, where SPI patterns are not recognizable anymore. It is relevant to note that the cessation SPI pattern became not reproducible, even though the same current input was applied to VPd (which provides inhibition to DP-PCN). These results could be expected, as the network lost any feedback from CN, a characteristic that was suggested to be responsible for the EOD production rhythms. As the corollary discharge pathway is not a direct connection between CN and VPd, providing inhibition from CN to DP/PCN, or even activation from DP/PCN to VPd may be another way to model the same corollary discharge pathway and assess the relevance of feedback loops in the electromotor command network. Nevertheless, none of these connections (between CN and PCN/DP or PCN/DP to VPd) has been reported.

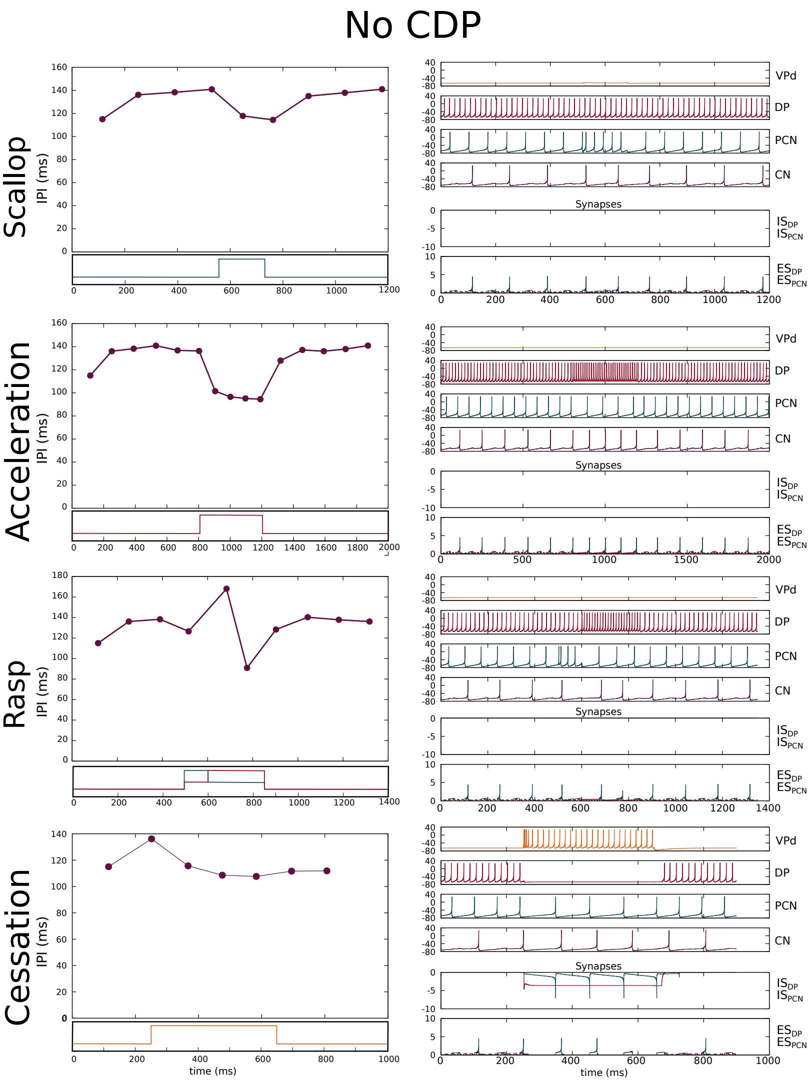

Supplementary Figure 7.1: Simulation of the four SPI patterns in the best fitting model without corollary discharge pathway. The results are depicted in two columns: the first one (left) shows SPIs resulting from the simulations and the second one (right) shows the nuclei voltages (top) and synaptic currents (bottom).

8 NO PCN PATHWAY

To address how the topology of the model and, more specifically, the presence of two different synaptical pathways affects the diversity of SPI patterns showed by the mormyrids electromotor system, one of these two pathways was removed (more precisely, the one corresponding to the PCN nucleus). GA was then applied to fit synaptical parameters (for the rest of synapses) to both recorded and synthetic data. The best result obtained in our tests is depicted in Fig. 8.1, where SPI patterns are not recognizable anymore. This reinforces the importance of the two synaptical pathways in the model's topology to reproduce the variability of SPIs showed by the electromotor command network.

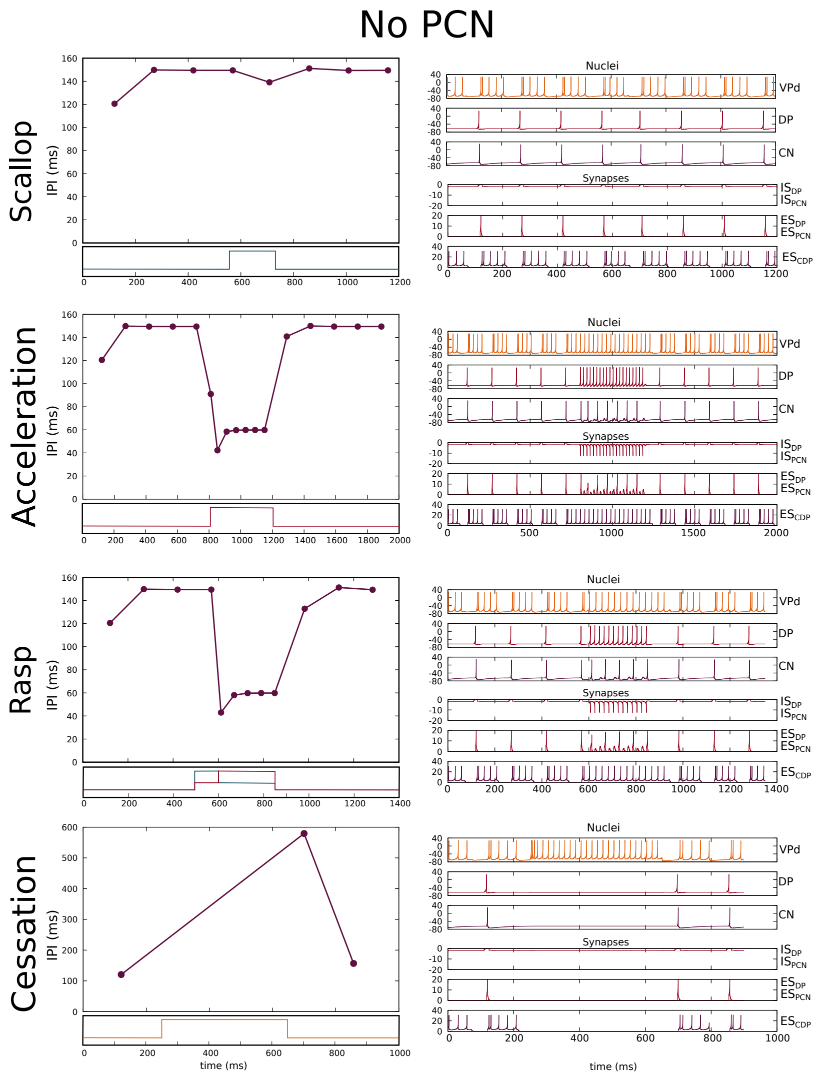

Supplementary Figure 8.1: Simulation of the four SPI patterns in the best fitting model without PCN nucleus pathway. The results are depicted in two columns: the first one (left) shows SPIs resulting from the simulations and the second one (right) shows the nuclei voltages (top) and synaptic currents (bottom).

9 NOISY INPUTS

We performed a set of simulations for the three configurations of the model with noisy inputs. Gaussian noise with zero mean value and 2 mA of standard deviation was applied in all the inputs of the model. This standard deviation implies a noise between 12.5% and 50% of the clean input signal, depending on the neuron and its activation in each pattern.

The SPI simulation results are depicted in Fig. 9.1.

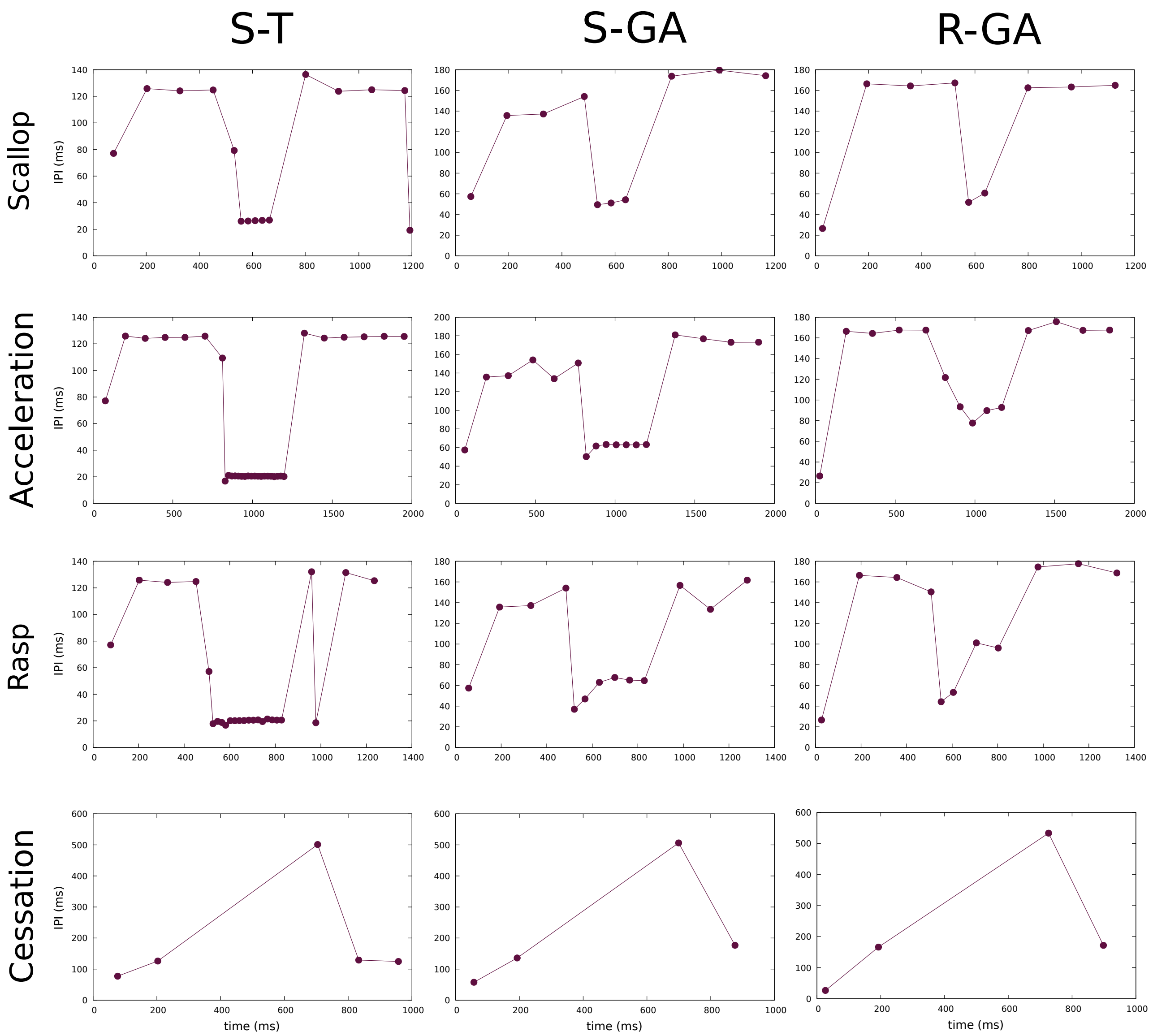

Supplementary Figure 9.1: Simulation of the four SPI patterns after adding Gaussian noise (mean = 0 mA; std = 2 mA) to inputs. All SPI patterns are still recognizable for configurations S-GA and R-GA. S-T is the least robust configuration, and, for instance, rasps lost their internal temporal characteristics.

It can be seen, comparing these results with those from Fig. 4, that, although distorted, all SPI patterns are still recognizable for configurations S-GA and R-GA. Regarding S-T, the rasp repeatedly show a short

IPI during its final resting phase as a result of a noisy input that makes PCN to fire. Although not always occur, there is a high probability of seeing this kind of scallop just after a rasp in this configuration.

Regarding R-GA, all the internal SPI characteristics were maintained:

- In scallops, a sudden drop to the shortest IPIs followed by an almost immediate recovery.
- In accelerations, a series of almost regular short IPIs (all of them above the minimum IPI in scallops).
- In rasps, a sudden drop followed by an acceleration-like tail of regular IPIs.
- In cessations, a long pause (of hundreds of ms) in EOD production.

10 ROBUSTNESS ANALYSIS DISAGGREGATED BY SPI PATTERN

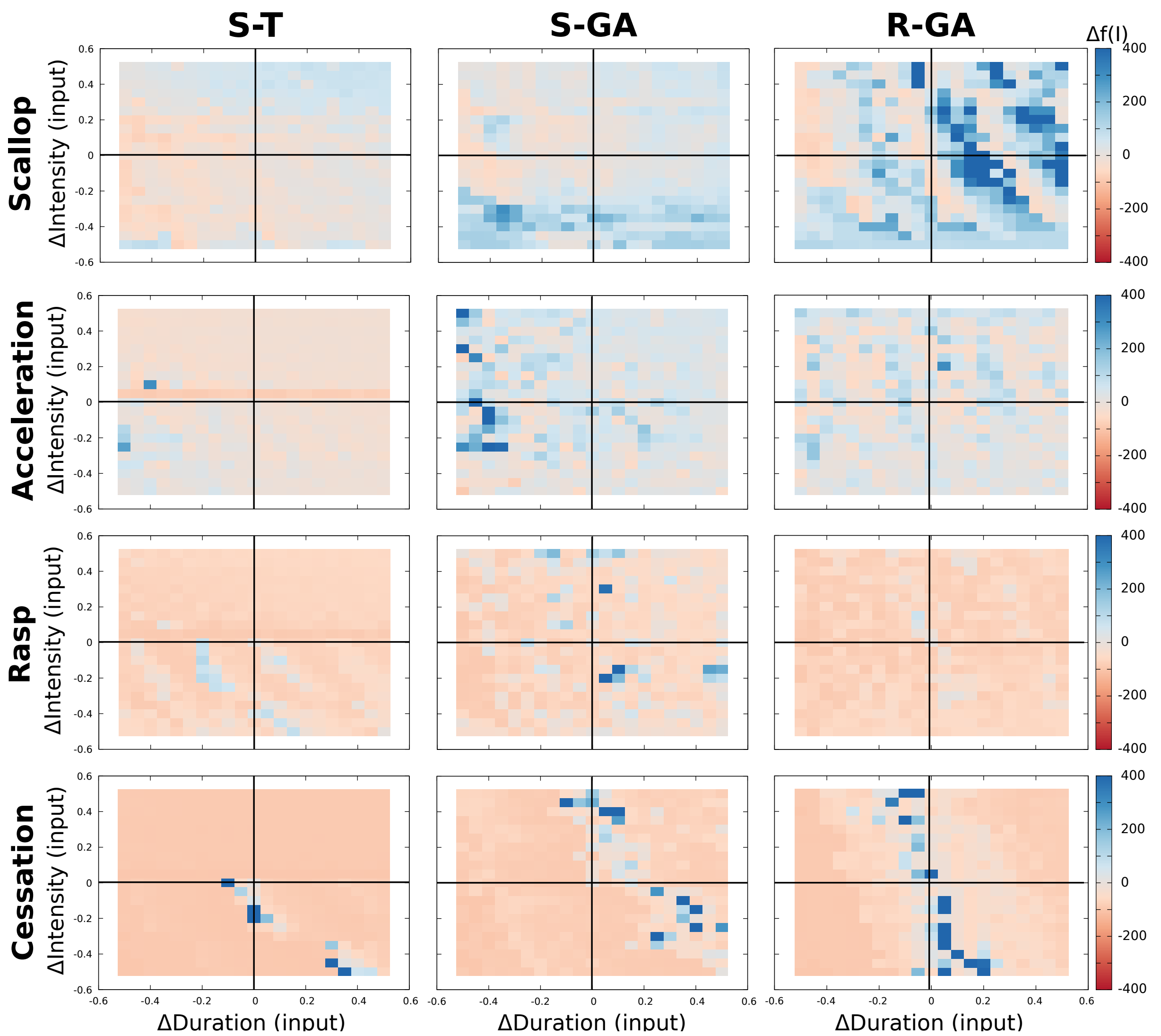

Supplementary Figure 10.1: Deviation from the f_P^0 (reference fitness value) of each model configuration (S-T, S-GA and R-GA) modifying default current input intensity ($\mathrm{In}_p^0(n)$) from $-0.5 \cdot \mathrm{In}_p^0(n)$ to $0.5 \cdot \mathrm{In}_p^0(n)$ in steps of 0.05, and also modifying default current input duration ($t_p^0(n)$) from $-0.5 \cdot t_p^0(n)$ to $0.5 \cdot t_p^0(n)$ using the same step. The color scheme is the same as the one described in Fig 5. See Fig. 6 for a representation of the IPIs (mean and variance) resulting from worse (light yellow), similar (orange) and better (dark red) fitness results in R-GA.

To discard overfitting to any of the SPI patterns, the robustness analysis was disaggregated by pattern. Results are shown in Fig. 10.1 (rightmost column). No relevant drops in the fitness results stand out in these panels for variations of up to 50% in the intensity and duration of the predefined stimulation inputs. These results held true even for larger changes in the duration or the intensity of the model inputs (see supplementary section 11 for details about the sensitivity of the robustness analysis). Quite the opposite, better evaluation results (depicted in dark red) were usually obtained, for example, in R-GA scallops, especially for increases in the input duration. These results show that patterns are not hard-coded to simulation timings (which were defined in tables 4, 5, 6, and 7).

For comparison, we also depict in Fig. 10.1 the robustness results of the other two configurations of the model (those adjusted to synthetic patterns: S-T, S-GA). S-GA and S-T configurations showed strong robustness to changes in the stimulation inputs (both in terms of intensity and duration), which is coherent with synthetic target patterns, showed less richness and thus, were more easily reproduced. S-T moderately improved the fitness results in accelerations and cessations when an increase in the duration of the inputs was compensated with a decrease in the input duration. S-GA, in general, showed greater robustness to input variability, maintaining or improving fitness in all SPI patterns.

11 SENSITIVITY OF THE ROBUSTNESS ANALYSIS

The robustness analysis presented in the paper was extensively used to test the presence of instability while tuning the model. Instability was one of the main reasons for adopting an automatic method for fine tuning the model and addressing the importance of synaptic properties for reproducing the network dynamics. In a few cases, we observed that slight changes in synaptic parameters resulted in significant drops in the model performance, both in terms of the fitting value and the quality of the resulting SPI patterns. For example in S-T, a critical case is changing the beta parameter in IS_{PCN} from 0.005 to 0.006 in S-T, which leaded to a $\Delta f = -371.4$ ($\Delta f(\text{scallop}) = -0.1$; $\Delta f(\text{acceleration}) = -322.7$; $\Delta f(\text{rasp}) = -758.2$; $\Delta f(\text{cessation}) = -3729.2$; see SPI pattern output in Fig. 11.1).

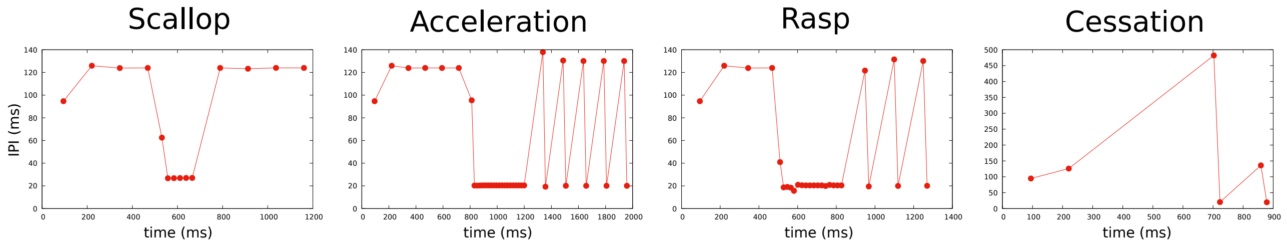

Supplementary Figure 11.1: Simulation of the four SPI patterns after a critical slight change in S-T synaptic parameters (changing only the beta parameter in IS_{PCN} from 0.005 to 0.006).

This result indicates that there is enough sensibility in the relative fitting value used in the robustness test, as significant drops in Δf take place due to relevant changes in the temporal structure of the resulting SPI patterns (as one can see in the figure above, cf. the objective patterns in Fig. 2 of the main text).

Regarding changes in the simulation parameters (duration or intensity of the stimulus presented to the model, see Fig. 5 of the main text), they also lead to losses of SPIs internal structure but not in such a critical way as it happens when we change the synaptic parameters as long as the proper nuclei are stimulated, as shown in Fig. 11.1.

We have considered a 50% change in both intensity and duration of the stimuli sufficient to address overfitting. Note that the test still shows $\Delta f < -100$ for certain cases. Nevertheless, as shown in Fig. 7 of the main text, SPI pattern internal structure is maintained. For the sake of clarity, we also present here an example case with extreme increments: $\Delta\text{duration} = 100$ and $\Delta\text{intensity} = 100$. Results in the fitting function are $\Delta f = -116.4$ ($\Delta f(\text{scallop}) = -32.8$; $\Delta f(\text{acceleration}) = -9.3$; $\Delta f(\text{rasp}) = -455.8$; $\Delta f(\text{cessation}) = -20500.6$) and the following SPI pattern output:

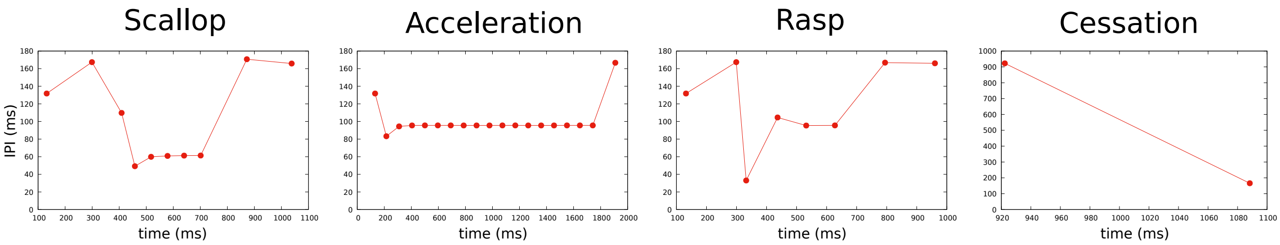

Supplementary Figure 11.2: Simulation of the four SPI patterns in a larger change of R-GA simulation parameters ($\Delta\text{duration} = 100$ and $\Delta\text{intensity} = 100$).

One can see that cessation loses its internal structure due to a loss of initial spikes (inhibition started early after its first spike), and the scallop starts to look like an acceleration due to its duration. Thus, the temporal structure of some patterns degenerates, in others, their temporal structure begins to transform, and in others, their temporal structure is maintained when large increases of stimulation in the nuclei are applied. So overfitting to intensity and duration of the simulation parameters can still be discarded.

In conclusion, the robustness test is capable of detecting strong changes in the temporal structure of SPI patterns. The most serious drops in the relative fitting value occur with changes in synaptic parameters, not so much due to changes in the duration or intensity of the inputs.

12 CONTINUITY BETWEEN S-T AND S-GA CONFIGURATIONS

The provided S-T and S-GA configurations are different well-tuned solutions to synthetic data. We address here the continuity of the fitting value between S-T and S-GA using a model configured with the mean value of each synaptic parameter between both configurations. The results of the model obtained from such configuration yield $\Delta f = -116.4$ ($\Delta f(\text{scallop}) = -32.8$; $\Delta f(\text{acceleration}) = -9.3$; $\Delta f(\text{rasp}) = -455.8$; $\Delta f(\text{cessation}) = -20500.6$) relative to S-GA. The SPI patterns output is depicted in Fig. 12.1.

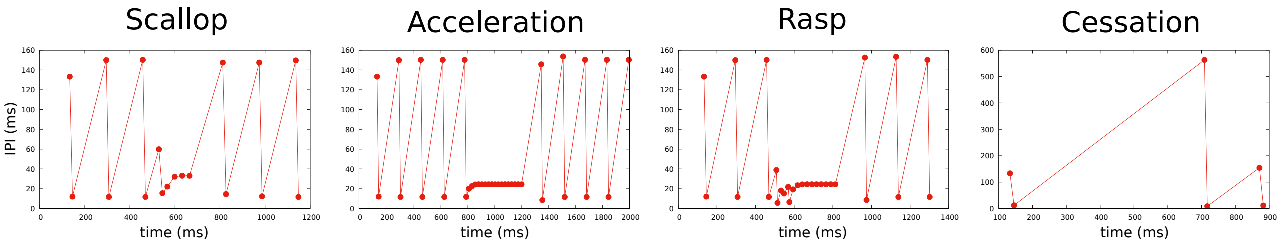

Supplementary Figure 12.1: Simulation of the four SPI patterns in the model obtained from the mean value of each synaptic parameter between S-T and S-GA.

This, together with the results of small changes leading to significant drops in fitting value, seems to indicate that S-T and S-GA do not define a continuous range of solutions, but that a delicate balance between synaptic intensity and duration is needed to succeed in reproducing the SPI patterns.

13 SOURCE CODE

All the software used to develop and run the model, as well as the model itself, is publicly available under an open-source license in a GitHub repository:

`https://github.com/GNB-UAM/electromotor-nmodel`

The repository includes:

- C++ code needed to run the model, which makes use of the *Neun* dynamical-systems library[3].
- The genetic algorithm applied to tune the parameters of the model automatically, implemented using GAlib[4].
- Examples of the recorded and synthetic patterns used as targets to tune the model.
- Finally, detailed instructions to compile and use the software.

REFERENCES

Caputi, A. A., Carlson, B. A., and Macadar, O. (2005). Electric organs and their control. In *Electroreception* (Springer). 410–451

Carlson, B. A. and Hopkins, C. D. (2004). Stereotyped temporal patterns in electrical communication. *Animal Behaviour* 68, 867–878

Destexhe, A., Mainen, Z. F., and Sejnowski, T. J. (1994). An efficient method for computing synaptic conductances based on a kinetic model of receptor binding. *Neural computation* 6, 14–18

Izhikevich, E. M. et al. (2003). Simple model of spiking neurons. *IEEE Transactions on neural networks* 14, 1569–1572

Lareo, A., Forlim, C. G., Pinto, R. D., Varona, P., and Rodriguez, F. (2016). Temporal Code-Driven Stimulation: Definition and Application to Electric Fish Signaling. *Frontiers in Neuroinformatics* 10, 41

Lareo, A., Varona, P., and Rodriguez, F. B. (2018). Evolutionary Tuning of a Pulse Mormyrid Electromotor Model to Generate Stereotyped Sequences of Electrical Pulse Intervals. In *Artificial Neural Networks and Machine Learning – ICANN 2018*, eds. V. Kůrková, Y. Manolopoulos, B. Hammer, L. Iliadis, and I. Maglogiannis. Springer (Cham: Springer International Publishing), 359–368. doi:10.1007/978-3-030-01421-6_35

Moller, P., Serrier, J., and Bowling, D. (1989). Electric organ discharge displays during social encounter in the weakly electric fish Brienomyrus niger L.(Mormyridae). *Ethology* 82, 177–191

[3] https://github.com/angellareo/neun

[4] http://lancet.mit.edu/ga/
